# European road transport policy assessment: a case study for Germany

**DOI:** 10.1186/s12302-022-00663-7

**Published:** 2022-09-15

**Authors:** Michael Schulthoff, Martin Kaltschmitt, Christoph Balzer, Karsten Wilbrand, Michael Pomrehn

**Affiliations:** 1 Institute of Environmental Science and Energy Economics, Hamburg University of Technology, Eißendorfer Straße 40, 21073 Hamburg, Germany; 2 Shell Germany GmbH, Suhrenkamp 71-77, 22335 Hamburg, Germany

**Keywords:** Transport policy, Evaluation, Environmental policy instruments, Road transportation, Regulation, Policy assessment

## Abstract

In the EU, the transport sector is the only sector with increasing GHG emissions compared to 1990. While harmful emissions have decreased due to successful regulation, transport performance, fossil fuel consumption and thus CO_2_ emissions have continued to increase, despite powertrain efficiency improvements. Meaningful regulation, which can be market-based (MBI) and non-market-based (NMBI) by nature, is needed to meet climate targets. To understand the mechanisms, effects and limitations of MBI and NMBI, this study investigates and evaluates selected regulations in the German road transportation sector until 2020. Therefore, this study identifies, describes, and categorizes environmental policy instrument types. Based on this step, selected instruments in the road transportation sector are identified by their type and implemented policies are described and assessed. Furthermore, an assessment methodology is developed to evaluate and score target achievement, cost-efficiency and practical feasibility by linking the outcomes of instruments to its goals. Based on the findings of this assessment, conclusions and recommendations are developed and discussed. Finally, results and general properties of policies and their type of instruments are extrapolated, and general statements about market and non-market-based instruments in a broader context for future regulation and market designs are projected. The study discovers that fuel producers and distributors, vehicle manufacturers and sellers are directly regulated by non-marked-based instruments, despite the EU Emissions Trading Scheme (ETS). On the customer side, primarily market-based implemented except for low-emission zones, which are direct regulations. The study finds that holistic representation and realistic internalization of external effects in a market is complex and will never be complete. Still, sufficient representation can be enough to drive transformation in the transport sector. The CO_2_ price itself is not sufficiently representing the consequential costs of climate change induced by road transport, but it helps to make low-carbon alternatives economically viable. Overall, the study finds that most implemented regulations in the German road transport sector were successful in relation to their goals.

**Supplementary Information:**

The online version contains supplementary material available at 10.1186/s12302-022-00663-7.

## Introduction

Burning fossil hydrocarbons releases energy by forming, among other airborne substances, carbon dioxide (CO_2_). This combustion product is released as waste into the atmosphere. The vast amount of CO_2_ produced by human activities throughout the last 150 years is slowly causing an imbalance in the earth´s climate system, resulting in climate change. This change can create significant threats to nations, societies and—apart from other environmental damage—human existence. Therefore, the global society has decided to limit additional fossil fuel-based CO_2_ emissions to keep global warming well below 1.5 °C relative to pre-industrial times.

As a consequence, societies need to transition toward a more sustainable and CO_2_-neutral energy system. A wide spectrum of very diverse policy instruments (EPI) has been developed in recent years to address the various sources of climate change and to move our society toward less GHG emissions. These policy instruments are based on various principles—such as polluter pays or prevention principles—and differ in their way of operation.

While within the European Union (EU), the power and industrial sector decreased their CO_2_—and other harmful—emissions during the last decades, the road transportation sector emission levels have steadily increased. But also, road transportation capacity and individual passenger mobility demand rose during this timeframe. Therefore, improvements in efficiency have been fully compensated in terms of the overall CO_2_ emissions from this sector. In addition, projections show that especially the commercial road transportation demand will continue to grow within the EU.

To address this development and to force the transportation sector to contribute to CO_2_ mitigation, well-designed environmental policy instruments need to be applied to accelerate the shift to low- or even zero-carbon transport technologies and behaviors. Within this context, assessing such instruments already applied within the transportation sector within the EU is fundamental.

The present study identifies, describes, and categorizes environmental policy instrument types (see Additional file 1). Based on this step, applied instruments in the road transportation sector are identified by their type and implemented policies are described and assessed. Therefore, an assessment methodology is developed to evaluate and score target achievement, cost-efficiency and practical feasibility. Based on the findings of this assessment, conclusions and recommendations are developed and discussed. Eventually, results and general properties of policies and their type of instruments are extrapolated, and general statements about market and non-market-based instruments in a broader context for future regulation and market designs are projected.

## Assessment of selected policies

### Brief profile of road transportation in Germany

Road transport has a significant impact on the climate. The German transport sector emitted 164 Mil. tons CO_2_eq in 2019 (pre-COVID), representing 20% of German GHG emissions. 160 million tons of CO_2_eq were produced alone by the road transport sector (see Additional file 1: 10 and 11). Compared to 1990, the transport sector has shown no total reduction in GHG emissions, while its share increased by 7% of total German GHG emissions [74]. The final energy demand (FED) from transport slightly decreased from 655 TWh in 1990 to 638 TWh in 2019, a reduction of 2.6%. In 2019, the road transport sector represented 592 TWh representing 93% of transport sectors FED (see Additional file 1: 12 and 13) [38, 64]. The number of registered passenger cars steadily increased from 30,6 Mil. in 1990 to 47,1 Mil. Cars in 2019 (2021: 48,3 Mil.) in Germany [66]. Furthermore, the average power of cars increased by 66% between 1995 and 2019. Cars became 11% heavier due to a rising share of sport utility vehicles (SUV) [47]. In 2019, 98% of cars were fueled by petrol (66%) and diesel (32%), while alternative powertrains were only 2% (see Additional file 1: 12) [1, 56]. More supporting statistics are shown in Additional file 1 in chapter 3. The data indicate that Germany's road transportation sector has not moved toward climate neutrality since 1990. Similar behavior can be observed in other European countries. Therefore, a bouquet of various environmental policy instruments was implemented to reduce the transport sector's impact on tackling the road transport sector. Further policy and revisions are yet to come due to the “fit for 55” package.

### Selected policies

**Fig. 1:**
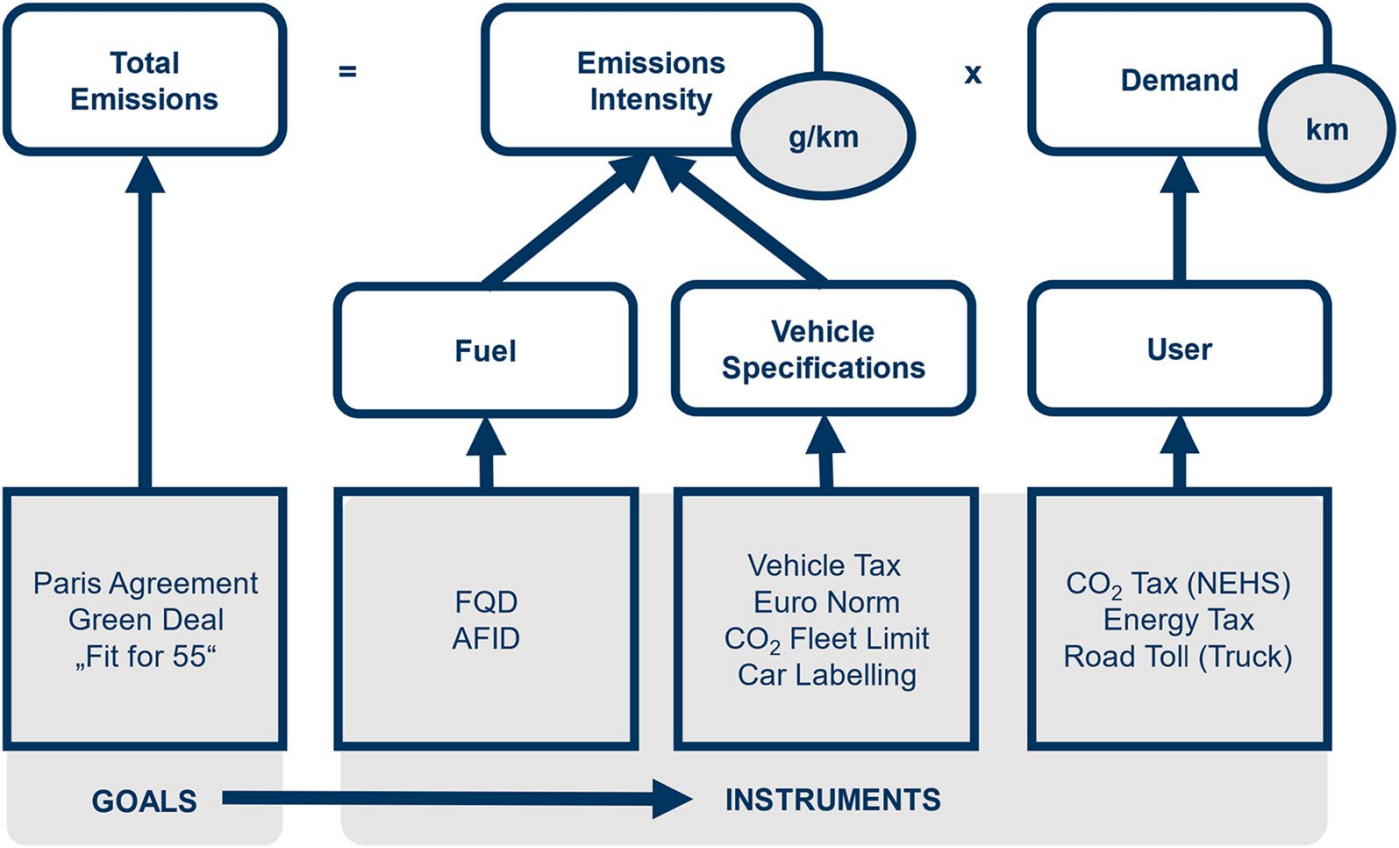
Relation of policy instruments in 2020 in road transport according to the total emissions

Different environmental policy instruments have been applied to realize environmental protection within this sector [55]. Some instruments focus on mitigating relative or specific emissions or performance standards, while others aim to reduce the overall absolute emission amount. Figure 1 places the regulations applied in the context of total emission reductions. Total emissions are a product of the total fuel demand and the emission intensity of the respective vehicles. Therefore, reducing total emissions can be achieved by mitigating one or all of those factors. While the fuel demand depends on user behavior, the emission intensity is related to the fuel type and the respective vehicle specifications. Each of these factors can be addressed with different environmental policy instruments to meet the set goals.

For example, the German government regulates—partly based on EU Directives—the transport sector by a spectrum of environmental policy instruments with overall 42 policies in 2021 [53]. Table 1 summarizes the central policies in road transportation in Germany. Non-market-based instruments are mainly applied for minimizing harmful emissions from fuels and exhaust gases (burning products). Market-based instruments are additionally used for emission reductions, e.g., by vehicle taxation—based on vehicle properties—and consumption of fuels. These instruments are listed by their objective downward the supply chain. Notably, non-market-based instruments are preferably applied to producers and distributors, whereas market-based instruments are chosen for consumer regulation. From a lifecycle assessment standpoint, cradle-to-gate impacts are regulated by non-market-based instruments, as markets do not fully internalize all relevant (external) effects for a holistic assessment. ETS certificates required to produce fuels and vehicles are not mentioned, because this is not a road transportation-specific mechanism.

**Table 1:** Regulatory framework for commercial vehicles and passenger cars

Designation	Orientation	Category	EPI-Type	Regulated objective
Fuel quality directive	Fuel Producer/distributor	NM	Technology mandates	Reducing GHG intensity of fuels
Euro norm	Vehicle producer	NM	Performance/technology standard	Limit harmful non-CO_2_ emissions
Directive on mobile air conditioning systems	Vehicle producer	NM	Performance Standard	Refrigerant of light vehicles
CO_2_ fleet limits	Vehicle producer	NM	Performance Standard	Limit harmful CO_2_ emissions for new PC and CV (after 2020 also for HDV)
Car energy labeling	Vehicle producer/seller	NM	Labeling	PC: Visualization of energy class
EV purchase incentive	Consumer	M	Subsidy	EV Incentive for PC and CV
Vehicle ownership tax	Consumer	M	Tax	Vehicle-related tax
Energy tax	Consumer	M	Tax	Fuel consumption
CO_2_ tax (NEHS)	Consumer	M	Tax	Fuel consumption
Low emission zones	Consumer	NM	Performance Standard	Harmful emissions limits for cities
Truck toll	Consumer	M	Tax	Toll for trucks > 7.5t per km

*M* market-based, *NM* non-market-based, *PC* passenger car, *CV* commercial vehicles, *EV* electric vehicles

Thus, CO_2_ emissions are regulated by different instruments in Germany. On the one hand, CO_2_ is controlled passively by preferences of the fuel, CO_2_ fleet limits and car labeling. On the other hand, market-based instruments policed by vehicle, energy and CO_2_ taxes are based on the polluter-pays principle. Consequently, the transportation sector has a higher implicit CO_2_ price compared to the explicit costs for CO_2_ within the ETS [45].

### Evaluation criteria

Environmental policy instruments affect different areas of the economy, environment, and society. Therefore, the evaluation of such instruments is based on the assessment of target achievement, cost-effectiveness, and practical feasibility [45], adopted from the EU environmental policy evaluation. Relevant studies and data sets are investigated for each criterion to emphasize key performance points.

- **Target Achievement** measures whether and to which degree a policy achieves the set objectives or goals. A policy measure serves different objectives, although a hierarchy of the different objectives is not necessarily discernible. Emissions reduction is often the explicit or implicit goal of such an environmental policy instrument. Explicit regulations target the issue directly, while implicit policies target the reduction by regulating an objective related to emissions. Results of target achievement can be different: Policy outcomes (e.g., laws and directives issued), outputs (e.g., the share of biofuels), and impacts (e.g., mitigation of risks resulting from climate change).
- **Cost-efficiency** relates the inputs (cost) and results (effects) of policy intervention. It provides an economic metric for efficiency and helps to compare different instrument types [45]. The efficiency can be related to microeconomic cost (e.g., implementation cost for technology from a producer/consumer perspective) or macroeconomic cost (e.g., the social cost of health issues induced by air pollution). Furthermore, micro- and macroeconomic costs can be compared to each other. Efficiency is divided into its static and dynamic components. Static efficiency reflects the most favorable avoidance variant at a given time (low-hanging-fruit principle; see Additional file 1: 1.6). The dynamic efficiency considers an intervention over a certain period. As a future-oriented criterion, dynamic efficiency necessarily contains an element of uncertainty. Since the cost and profitability of new technologies cannot be forecasted with certainty, it will also be difficult to accurately determine the efficient level of investment in low-carbon technology innovation and diffusion. To assess the cost-effectiveness of policy measures, the cumulative net costs discounted over time need to be considered. In a macroeconomic framework, this also includes the opportunity costs of investment [11, 45]. In this analysis, the cost-efficiency represents the magnitude of the cost impacts of a measure in relation to their achieved targets, based on studies made for each applied instrument.
- **Practical feasibility** is a very heterogeneous criterion set. It summarizes other evaluation criteria associated with the instruments and the field applied. The various criteria thus refer to the difference between policies as designed on the drawing board and their actual implementation in practice. Therefore, they are grouped under the heading of feasibility. The spectrum of possible sub-criteria can include administrative implementation (e.g., reporting and review effort), unintended side effects, political acceptability, legal and institutional feasibility, flexibility, risk, and uncertainties [45].

### Scoring

Each environmental policy instrument reviewed with the criteria defined in Sect. 2.3 is scored in a three-step range. The applied spectrum is presented in Table 2. As the criterion practical feasibility is heterogeneous, the scoring is related to its impact on the relevant sub-criteria. The evaluation takes place from the regulator's perspective or with a focus on the impact on society. For this purpose, the micro-economic costs (private-sector) incurred are placed in context with macroeconomic costs. Since the objectives of the environmental policy instruments (EPI) differ by their type, no overarching quantified assessment can be made here, either on an absolute or a relative scale. In some cases, several EPIs pursue the same reduction targets and changes, but the respective share of each EPI cannot be reliably determined. For example, CO_2_ tax and energy tax both directly aim at a decline in demand and, therefore, indirectly at emissions reduction.

**Table 2:** Scores and description

Score	Key	Effectiveness (Goals)	Efficiency
High		Mostly achieved	Mostly efficient
Intermediate		Partly achieved	Partly efficient
Low		Hardly/not achieved	Mostly inefficient

Moreover, the technological development (implied by the market pressure) toward higher efficiency, lower fuel consumption—and thus low emissions—is naturally brought about by lower operating costs. Reduction of CO_2_ is thus automatically enforced by the market if fuel prices are sufficiently high. In contrast, reducing harmful emissions—covered by Euro standards—does not entail any cost savings for the user.

## Evaluation profiles of policies in Germany´s road transportation sector

In the following, the applied instruments in the German road transportation sector are evaluated. Therefore, each instrument (shown in Table 1) is described, its targets are identified, the performance related to the criteria (shown in 2.3) is evaluated, and each performance key point is scored (based on Table 2).

### Fuel quality directive (FQD)

#### Description

The Fuel Quality Directive (FQD) and its amendments establish environmental requirements for gasoline and diesel fuels to reduce their emissions of air pollutants issued by the EU. The FQD combined with the Renewable Energy Directive (RED II) set standards for environmental emissions. The RED II targets to increase the share of renewable energy consumption mix by 32% by 2030, and the member states require fuel suppliers to include a minimum of 14% renewable energy consumed in rail and road transport. Besides the Fuel Quality Directive (FQD) Implementing Directive, the ILUC Directive and the “Winter Package” contain further the regulations for fuel suppliers [25].

The targets of the fuel quality directive until 2020 can be summarized as follows [25, 33]:

1. Contribute to enhanced air quality
2. Contribute to greenhouse gas (GHG) reduction and biofuels sustainability. Reduction of the average life cycle GHG intensity of transport fuels brought into the market by a minimum of 6% by the end of 2020 (Indirect land-use change (ILUC) is not taken into account)
3. Reduce impacts on health and environment from transport fuels
4. Reduce greenhouse gas and air pollutant emissions from the transport sector
5. Ensure proper functioning of engines and after-treatment systems
6. Guarantee the quality of petrol and diesel
7. Ensure a single market for fuel (setting minimum standards for selected specifications)

#### Performance and score

In the following, the performance of key aspects for the selected criteria is summarized and shown in detail in Table 3.

- Most of the targets set for the FQD have been achieved, although the total GHG reduction targets have only been met partly or not at all. For example, only 62% of the life cycle GHG intensity reduction goal of 6% has been completed within the EU (excluding ILUC). Furthermore, no absolute CO_2_ reduction was achieved by the FQD; instead, CO_2_ emissions increased due to an increased demand for fuels. However, the FQD does not directly handle fuel demand (see Fig. 1). The CO_2_ intensity reduction of 3.1% by 2020 has led to a less pronounced increase in emissions and is, thus—despite missing the target—to be assessed as positive and in line with the policies overarching goals [15, 22].
- The cost-efficiency assessment is primarily based on an evaluation of the European Commission from 2017. As the FQD is the only instrument directly aiming at the GHG intensity of fuels, the related costs cannot be compared and set into context with similar measures. The RED indirectly aims to reduce GHG intensity by setting goals for renewable energy shares. The costs mainly occur at a micro-economic level, which means at the industry or consumer level (unless the monitoring costs), while the benefits are at a macro-economic level. The occurring costs for the fuel distributors are at a much lower magnitude than the benefits for society due to avoided damage costs from harmful emissions.
- The FQD is considered practically, as the intervention is mostly coherent with existing measures, except for inconsistencies of biofuels with the Renewable Energy Directive (RED). Without the FQD, promoting a single market for producers could not have been ensured.

**Table 3:** Performance and score of fuel quality directive

Performance		Score	Refs.
*Target achievement*			
(1)	Reduction of NO_x_, Pb, SO_x_, PM and PAH emissions from transport vehicles significant		[15, 46]
(2)	GHG reduction goal: 3.7% (62% of goal), respectively 2.1% (35% of goal) incl. ILUC Upper limits for bioethanol (10%) and FAME (7%) Lack of harmonization of biofuel mandates Implementation of multiple counting of alternative fuels to promote certain fuels		[15, 22]
(3)	Combination with other measures (e.g., Low-emission zones (LEZ), Euro-Norms) Historical data shows significant air pollutant reduction		[69, 73]
(4)	No reduction of total CO_2_ emissions due to higher demand (see Additional file 1: 11) Reduction of specific fuel GHG emissions by 3.7% Reduction of harmful emissions (see target (1))		[15, 71]
(5)	Proper function of engines ensured		[15]
(6)	Overall quality is guaranteed according to CEN standards EN228 and EN590 Petrol: majority placed on the market in the EU is compliant with Annex I specifications (almost 100%) Diesel: the majority is in accordance with Annex II The introduction of Euro IV was only possible after the regulation came into force		[46]
(7)	A single market was mostly ensured by defining minimum standards for the quality of fuels and the technical compatibility of these of these fuels with internal combustion engines and after-treatment equipment But bioethanol regulations were uneven across EU		[15] [46]
*Cost-efficiency*			
Desulphurization: 2001–2011 cumulative benefits of 197 Mil. € per refinery			[15, 22]
Estimated avoided damage cost: 695 Mil. € for a reduction in SO_x_, and 8,611 Mil. € (damage cost functions) for a reduction in NO_x_ for EU28 over the period 2009–2013			[15, 22]
Member States: Monitoring and reporting costs 173,000–650,000 € per year—comparatively low administrative overhead			[15]
Fuel suppliers: €202 million cumulative costs per refinery over 2001–2011 (51% corresponds to investment costs and 49% to operational costs)			[15]
Many petrol vapor emission derogations			[32]
The significant benefits of the FQD outweigh all these costs			[15]
Engine and emissions reduction performance benefits due to improved fuel specifications compatible with advanced engine standards			[15]
*Practical feasibility*			
FQD is considered coherent with the rest of the environmental legislation Issues regarding biofuels, provisions within the FQD itself and in relation to the RED The flexibility provided in Article 4 is used only to a very limited extent FQD is still considered relevant overall, and no article is classified as not relevant Restrictions that the FQD places on gasoline and diesel fuels remain relevant to ensure the health and environmental benefits of the FQD and to promote a single market for fuels within its scope Member States agree that the internal market could not be achieved without the Directive and that the Directive, therefore, has EU added value Reduce barriers due to fuel specifications harmonization The strong intra-EU market for fuel suppliers and vehicle manufacturers created			[15]

### Euro norms

#### Description

While the FQD regulates the fuel supply side, emissions standards are a common instrument regulating emissions from vehicles that manufacturers must abide by. In Europe, two types of emission standards are applied. Non-CO_2_ emissions are limited by the Euro norms for all road vehicles (as described in the following), whereas the CO_2_ fleet limits regulate CO_2_ emissions of passenger cars and LDVs [30].

The Euro norms are emission standards for passenger cars and commercial vehicles for motor vehicles and their specific replacement parts [25]. The policy aims to protect air quality, (indirectly) improve fuel economy, and encourage technological development and innovation [30]. The policy covers a wide range of tailpipe, evaporative and crankcase emissions. Thus, in-cylinder and after-treatment technologies were developed and implemented. Exclusively, harmful pollutants are regulated: carbon-monoxide (CO), non-methane hydrocarbons and total hydrocarbons (C_n_H_m_), nitrogen oxides (NO_x_), particulate matter (PM) and particle number (PN) [25].

Table 4 describes the evolution of Euro Norms for light-duty vehicles. Several technologies to mitigate pollution became mandatory, and as the test procedures changed, real driving emissions were included. Emissions are measured in the use of a vehicle on the road. The International Council of Clean Transportation (ICCT) provides further studies on the emission standards compliances costs for diesel LDV and HDV, including estimated costs for EURO 7 [59, 60].

**Table 4:** Evolution of emission standards for light-duty vehicles [41]

Emissions standard	Issued	Description
Euro 1	1993	Catalytic converter and electronic fuel injection mandatory for new registrations
Euro 2	1997	Limitations for petrol and diesel engines
Euro 3	2001	Obligation for On-Board Diagnostics (OBD) as an emissions diagnostics system for petrol engines (2004 for diesel)
Euro 4	2006	Adjustment of pollutant limits pave the way for diffusion of a particulate filter
Euro 5	2008	Limits tighten, the particulate filter becomes mandatory (for diesel)
Euro 6	2014	Stricter homologation and test procedures (WLTP and RDE), particulate filter for petrol cars and SCR for diesel cars
Euro 7	2025	Not issued yet (Legislative Proposal in 2022)—ammonia, methane (CNG fuels), and further regulations

As these tests are hard to reproduce on the road, real driving emissions were higher than those reported in laboratory tests, resulting in a confirmation factor by the EU that relates laboratory and real driving emissions [2]. The EU limits air pollution to mitigate cardiovascular diseases and premature statistical deaths. The World Health Organization (WHO) provides its Air Quality Guideline recommendations for outdoor and indoor pollution limits. The WHO defines the limits on several short- and long-term exposure studies investigating exposure–effect relationships. Lower limits are based on the so-called NOAEL (no observed adverse effect level), which shows no related effect of pollutants on health issues. Especially, particular matter shows a harmful impact on health with every pollution level and is associated with the particular size. Limit values result from a political balancing process, whereas health improvements versus feasibility and costs of the actions are weighted [63].

In 2017, WLTP and real-driving emission tests were introduced to improve testing procedures for passenger cars. New cars must pass these tests in real driving conditions and improved laboratory tests before receiving approval for European roads. Euro IV became mandatory for heavy-duty vehicles in 2013 [19]. Since 2019, newly produced trucks must determine and declare their CO_2_ emissions and fuel consumption with the latest version of the Vehicle Energy Consumption calculation Tool (VECTO) which was developed by the European Commission [34]. Furthermore, real driving emissions are tested by the verification testing procedure (VTP) to verify emissions and fuel consumption from July 2020 [30].

The implementation of the Euro standards generates additional costs due to the mandatory technologies to reduce emissions to achieve the targets. The costs were estimated by the ICCT in 2012 and are shown in Table 5. According to Euro 1, gasoline cars were required to switch from carburetor to electronic fuel injection systems and install catalytic converters, which are more complex and more costly. From Euro 2, the costs for emissions reduction in diesel LDVs to meet regulations were always higher than for gasoline LDVs. Especially the mandatory installation of particulate filters (Euro 4) and selective catalytic reduction catalysts (SCR) led to increasing costs for diesel vehicles. The average car price in 2012 was 30,000 $, which resulted in a cost-share of 1% for gasoline and 5—6% for diesel LDVs [42]. However, the introduction of Euro 6 made gasoline particle filter necessary for some cars to meet the regulations, which implied costs for the transition of Euro 5 to Euro 6 for gasoline cars. These—not mandatory, but for some cars necessary—particle filter costs are not estimated in the cited study.

**Table 5:** Incremental costs for LDVs meeting Euro Norms (in 2010 US dollars) [62]

Engine Type	Vehicle Class	Euro 1 (Baseline)	Euro 1 to Euro 2	Euro 2 to Euro 3	Euro 3 to Euro 4	Euro 4 to Euro 5	Euro 5 to Euro 6	No Control to Euro 6
Gasoline	4 cylinders Vd = 1.5 L	142	63	122	25	10	–	362
Gasoline	4 cylinders Vd = 2.5 L	232	3	137	15	30	–	417
Diesel	4 cylinders Vd = 1.5 L	56	84	337	145	306	471	1399
Diesel	4 cylinders Vd = 2.5 L	56	89	419	164	508	626	1862

*Vd* vehicle (engine) displacement

The targets of the Euro norms can be summarized as follows [13, 52, 77]:

1. Lower (harmful) air pollution from vehicles, improve air quality
2. Set fleet-wide performance standards (CO, NO_x_, SO_x_, C_n_H_m_, PM, PN) for the type of engine (diesel, gasoline) and vehicle type (car, LDV, HDV, motorcycles)
3. Indirect GHG emissions reduction (Euro 7 might include direct regulation of methane evaporation from CNG/LNG vehicles)
4. Implementation of representative and standardized laboratory test cycles and measure real driving emissions (mandatory for Euro 6)

#### Performance and score

In the following, the performance of key aspects for the selected criteria is summarized and broken down in Table 6.

- The Euro norms emissions standards achieved almost all of the set targets. It led to a significant reduction of harmful emissions (see SM2). Representative test cycles were implemented and constantly adjusted, but those test cycles do not fully represent real-world behavior. To account for this, real driving emissions (RDE) were introduced with the Euro 6 in 2014.
- The cost-efficiency is hardly evaluated. Each new regulatory level requires certain technologies to meet the targets or are mandatory (see Table 3), which creates additional costs for emissions treatment. On the one hand, the microeconomic costs for customers for LDVs are shown in Table 5 and are 1–6% of car price (related to an average car price of 30,000 $). On the other hand, the macroeconomic avoided damage costs are estimated at 8,611 Mil. € only by reducing NO_x_.
- Unified test cycles ensure comparability and make the policy practically feasible, but laboratory testing does not represent real-world emissions. Furthermore, real emissions are related to user behavior. This instrument has been implemented in several countries since 1990 (see SM9) [13].

**Table 6:** Performance and score of Euro norms

Performance		Score	Refs.
*Target achievement*			
(1)	Significant reduction of CO, NO_x_, SO_x_, C_n_H_m_, PM, and PN (see SM2) Several studies show a connection between early statistical deaths and harmful vehicle emissions Significantly fewer air pollution-related diseases in hospitals were found before the introduction of LEZ in 2012 due to better emissions standards Supported new low-emission technologies market entry, which leads to a reduction of emissions		[71] [13] [58]
(2)	EU-wide standard categories for newly registered vehicles (cars, LDV and HDV), independent of their gross vehicle weight Standards are revised and renewed due to the availability of technologies with lower emissions The base unit for cars and LDV is kilometers, while it is ton-kilometers for HDV		[30]
(3)	Indirect reduction of CO_2_, because decreasing consumption was crucial for harmful emissions mitigation (downsizing, turbocharger) CO_2_ value according to WLTP decisive for vehicle taxation		[41]
(4)	Testing standards for Europe (new European driving cycle—NEDC) and since 2017 worldwide harmonized light vehicles test procedure (WLTP) for cars Vehicles are optimized for the test cycle, not representing real driving emissions The introduction of WLTP and testing real-driving emissions (in-service testing) made a conformity factor necessary		[18] [21] [21]
*Cost-efficiency*			
Estimated avoided damage cost: 8,611 Mil. € (damage cost functions) for a reduction in NO_x_ for EU28 over the period 2009–2013			[15, 22]
A cost increase of mandatory reduction technologies of 1% for gasoline and 5–6% for diesel LDV (regarding average car price of 30,000 $ in 2012)			[42]
*Practical feasibility*			
Cold start emissions were not considered until 2021 Real-world emissions are not represented in driving cycles—in-service emissions since Euro VI The onset of effect: Euro IV took seven years (2014–2021) to reach a fleet share of 40% (see Additional file 1: SM 7)			[18] [21]

### CO_2_ fleet limit

#### Description

CO_2_ fleet limits define performance standards for specific CO_2_ emissions per base unit (distance [g/km] for passenger cars and commercial vehicles below 3500 kg and engine energy output [g/kWh] for heavy-duty vehicles). The base units are chosen differently, because those are related to the performance of a vehicle type. As the performance of passenger cars and light-duty vehicles is mobility itself, performance defined for heavy-duty is related to the transport capacity of a wide range of vehicle sizes. In 2020, the limits are 95 g_CO2_/km for cars and 147 gCO_2_/km for vans while targeting a 15% reduction for cars and vans in 2025 based on 2021 starting points [7, 28].

Table 7 summarizes the CO_2_ fleet limits for cars and vans. As the fleet limits become more restrictive, a technology shift is indirectly induced, because standard internal combustion engines (ICE) cannot meet the emerging requirements. ICEs can meet those goals if they are verifiable fueled (blended or fully) with low-/zero-carbon fuels, such as biofuels or synthetic fuels (E-Fuels). However, even if the verifiable use of low-carbon fuels is accepted for the CO_2_ fleet limits, the vehicles still have to meet the Euro norms regarding harmful emissions from combustion. At the time of conduction of this study, the discussion has not been solved. The EU annually sets specific emission targets for each manufacturer based on the EU fleet-wide average mass of the producer's new vehicles registered with a limit value curve [27].

**Table 7:** CO_2_ fleet limits reduction goals

Timeframe	CO_2_ reduction goals
2025–2029	15% compared to 2021 [7]
from 2030	55% for cars, and 50% for vans [27]
from 2035	100% for cars, and 100% for vans [27]

**Fig. 2:**
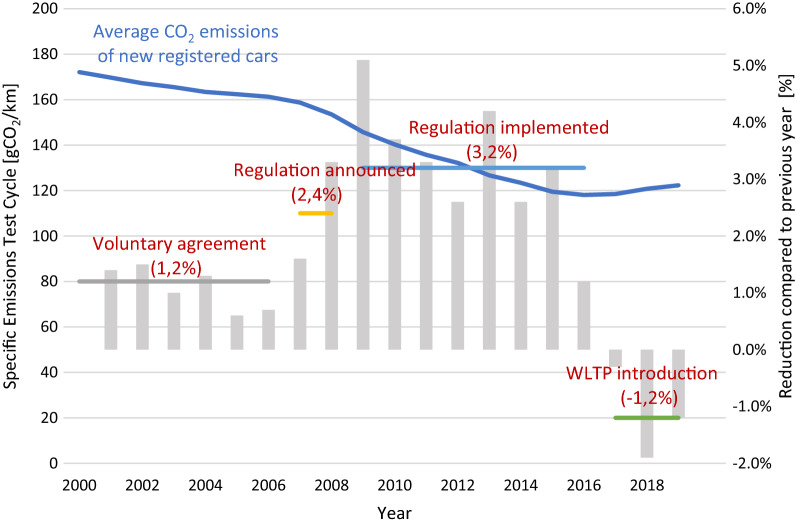
Specific CO_2_ emissions from test cycle (formerly NEDC), the introduction of WLTP test cycle, and previous year change (own figure based on [35]) from 2000 to 2019. Blue line: Average specific CO_2_ emissions of newly registered passenger cars

Figure 2 illustrates the specific CO_2_ emissions of newly registered passenger cars in Europe between 2000 and 2019. The blue line shows a constant reduction in gCO_2_/km during the NEDC test cycle. With the introduction of the WLTP test cycle, specific CO_2_ emissions increased slightly due to a more demanding and realistic driving cycle. Until 2006, no mandatory regulations were set, and the voluntary agreements of car manufacturers only led to a reduction of 1.2% of specific emissions on average per year. When the regulation was announced in 2006, the average reduction rate increased to 2.4% per year. In 2009, the regulation was implemented, which increased the reduction rate to 3.2% on average. During the voluntary agreement phase, the relative reduction between 2000 and 2006 was 6.3%. The announcement and implementation of the policy led to a decrease of 24.2% between 2006 (161 gCO_2_/km) and 2019 (122 gCO_2_/km). Therefore, the regulation successfully impacts the average specific emissions of newly registered cars.

Overall, the targets of the CO_2_ fleet limit can be summarized as follows:

1. Limitation of CO_2_ emissions from newly registered vehicles (cars and LDV)
2. No detriment to low-volume car manufactures

#### Performance and score

In the following, the performance of key aspects for the selected criteria is summarized and broken down in Table 8.

- The targets were achieved, as the CO_2_ fleet limits resulted in an overall reduction of 29.3% regarding the specific CO_2_ emissions of newly registered cars. Furthermore, low-volume manufacturers were not harmed by this regulation.
- According to Gibson et al. (2014), the CO_2_ abatement costs of this regulation were estimated between 32.4 and 39.8 €/tonCO_2_. Although the abatement costs are hard to evaluate due to the development cost of manufacturers and the cost of developing and running the test cycles (with each car model), the regulation is assessed as cost-effective. Furthermore, the penalties for non-compliance are high.
- Overall, the regulation is practically feasible as test cycles are standardized and reproducible. However, as laboratory test cycles on test facilities are used, they do not represent real-driving emissions (RDE). Therefore, RDE was added to WLTP. As RDE is much higher than the laboratory emissions, conformity factors were introduced.

**Table 8:** Performance and score of CO_2_ fleet limits

Performance		Score	Refs.
*Target achievement*			
(1)	130g_CO2_/km goal by 2015 was achieved		[35]
	Policies led to a rapid CO_2_ reduction rate in test cycles		[44]
	Led to implementation of innovations:		[44]
	•high-pressure fuel injection and auxiliary system improvement, hybrids, downsizing, 6-speed dual-clutch transmission and advanced EGR technology		
	•"super credits" for eco-innovation help to implement fuel-saving technologies, such as cylinder deactivation		
(2)	A low-volume manufacturer (responsible for fewer than 10,000 passenger cars or fewer than 22,000 vans newly registered per year)		[29]
*Cost-efficiency*			
A high penalty for exceeding limits (95 €/gCO_2_ per vehicle on fleet average) ensures compliance			[29]
Expected abatement costs between 32.4 and 39.8€/tonCO_2_ for passenger cars			[44]
*Practical feasibility*			
Self-commitment of car manufacturers for CO_2_ reduction failed. Therefore, the CO_2_ fleet limit was introduced in 2012			[7]
Test cycles constitute a significant issue of assessment—> switch from NETC to WLTP in 2017			[35]
Has the ability to compare different powertrains, but in 2020 only focusing on operative emissions			[44]
• Embedded emissions • Well-to-wheel emissions and lifecycle approach • Consideration of possible rebound effects • Impacts of emissions and other pollutants (Turbocharger and higher gasoline share lead to higher—and smaller—particle matter)			
Consumer decisions are still decisive—SUV share is rising (38% of newly registered cars in 2019 in EU)—limited effect on consumer choice Fig. 2			[35]
Onset of effect:			[7]
• Five years after implementation, 50% and after 10 years, 85% of the fleet were within the limit • Limit set in 2019 for 2030 will significantly form the fleet in 2040			
It has no effect on transport performance (kilometers driven), while passenger car transport performance is projected to increase by 10% by 2030			[7]
Issue of CO_2_-Pooling: Pure electric car manufacturers can sell their "super credits" to other car companies to lower their fleet emissions, as happened with Tesla, Fiat-Chrysler Automobiles and Honda in 2019. This CO_2_-pooling cooperation ended in 2021			[50, 67]

### Car labeling

#### Description

Car energy labeling ensures that relevant information about the vehicle is provided to consumers, containing fuel economy and CO_2_ emissions (classification from “A”—high to “G”—low) [26, 49]. The car energy labels classification is based on the WLTP test cycles (formerly NEDC) results.

**Fig. 3:**
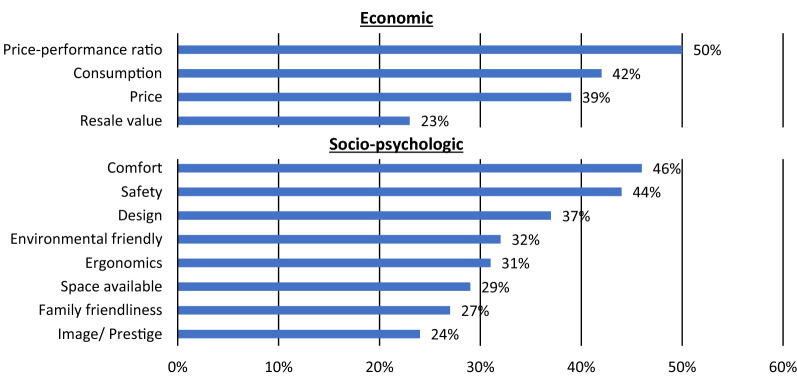
Selection criteria of car purchase from trends of car purchase 2021, based on [4]

The “Trends of car purchase report 2021” provides selection criteria of surveyed customers, shown in Fig. 3. The criteria are categorized into economic and socio-psychologic criteria. Price–performance ratio, consumption, and price are the main economic criteria for customers, while comfort, safety, and design are the leading socio-psychologic criteria. As environmental friendliness is in 8th place, it is less important for a purchase. Customers became more aware of emissions produced by their behavior during the last decade, but awareness and acceptance need to be further increased for less emitting cars [4]

The targets of car labeling can be summarized as follows:

1. Incentives for consumers to buy cars that use less fuel [49].
2. Visualize carbon dioxide and fuel efficiency [43].
3. The Car Labeling Directive (Directive 1999/94/EC) of December 1999 is a demand-side directive that should support manufacturers in meeting specific CO_2_ targets [26].
4. Labeling aims at environmental and behavioral economics by influencing customer's choices [14, 48].

#### Performance and score

In the following, the performance of key aspects for the selected criteria is summarized and detailly shown in Table 9.

- Car labels enhanced customer awareness due to different studies. However, major selection criteria for car purchases are price–performance ratio and comfort (1st and 2nd), while environmental friendliness is in 8th place. While consumption is another main criterion (4th place, see Fig. 3), car labeling might become more impactful due to rising CO_2_ taxes and a subsequent increase in fuel costs.
- The results show that car labels are cost-effective in reaching customers, impacting purchase behavior, and creating awareness.
- The practical feasibility of labels is also given, as they provide essential consumption and emissions information directly to the customer during the purchase process. Furthermore, car labels can help pave the way for a change to more environmental-friendly social norms.

**Table 9:** Performance and score of car labeling

Performance		Score	Refs.
*Target achievement*			
(1–4)	SUVs remain more attractive to customers—38% of newly registered cars in 2019 in the EU		[35]
	Customers´ awareness of fuel economy and CO_2_ emissions has increased steadily to 75% in member states since the directive was implemented		[24]
	Success of mandatory informative policies depends on the selection criteria of customers. The "Trends of car purchase 2021" report describes the main criteria, as shown in Fig. 3		[4, 49]
	The eco-friendly qualities play a secondary role for consumers, and other attributes such as price, performance and safety come to the fore		[4, 26]
	The evaluation shows no strong evidence of the supply of more efficient vehicles by manufacturers		[24]
	Experiments show that fuel economy and operating costs were better understood and influenced subjects' behavior when environmental friendliness was coupled with fuel economy		[26]
	No empirical evidence of a strong effect on the supply of more efficient vehicles by manufacturers to receive higher ratings		[14]
*Cost-efficiency*			
Monitoring and enforcement in the region of €10,000–100,000			[24]
Collection of information is a significant cost driver in some countries (the Netherlands and France) 70,000 €–90,000 € for the industry			[24]
Maintenance costs for (voluntary) online databases are in the range of 140,000 € to 240,000 € (Germany and UK)			[24]
Printing of guides accounts for a significant share of €30,000–60,000			[24]
Printing the labels, estimated at between €0.5 and €1 million for EU28			[24]
Benefits from car labeling are hard to link to quantifiable data			
*Practical feasibility*			
The difference in the application of car stickers and promotional materials between member states is creating confusion for consumers			[9]
Using labels such as colored energy labels and adding important information to raise awareness (e.g., operating costs)			[24]
Attitude-action gap: In most countries and for most types of products and services, consumers self-report that they want to make environmentally conscious purchases, but only in a minority of cases are these intentions translated into action			[14, 79]
Change in social norms: As environmentally friendly social values become more common, social normative nudges are likely to become more effective			[14]

### Vehicle tax

#### Description

Vehicle (ownership) tax sets common rules for the taxation of all kinds of motor vehicles. Therefore, the EU lays down general principles in the Treaties to which national provisions must adapt [23, 31]. As the role of CO_2_ emissions evolved, the taxation was adjusted several times during the last decades [75]. Furthermore, the calculation became more complicated as it differentiates between vehicle types and size criteria. Typical criteria are fuels (diesel, gasoline, alternative), type (car, commercial vehicles, motorcycles and more), emission standard (Euro Norm), registration year, CO_2_ emissions (WLTP), and gross vehicle weight (commercial vehicles) [55, 81]. This study exclusively evaluates the influence of vehicle taxation on passenger cars. The taxation system in 2021 and before for cars in Germany is shown in the Additional file 1 (Cars: SM 3, SM 4; HDV: SM 6).

The targets of the vehicle tax can be summarized as follows:

1. Create unrestricted state revenue for the state budget from private and commercial vehicle ownership [76].
2. Environmental steering effect on alternative and low-carbon powertrain technologies [76].

#### Performance and score

In the following, the performance of key aspects for the selected criteria is summarized and broken down in Table 10.

- The vehicle ownership tax achieved its tax revenue target due to a state income of 9.53 Bil. €. Regarding environmental effectiveness, the inclusion of CO_2_ emissions based on the WLTP test cycle (formerly NEDC) added an internalization of external effects from those emissions. It changed the displacement and fuel type-based tax system to a hybrid system. Furthermore, the former linear tax system was developed into a staggering tax system based on CO_2_ emissions to tax vehicles with higher emission values and thus heavier and larger vehicles more severely.
- The vehicle tax system is practical and flexible, as it allowed the change to a combination of a displacement- and a CO_2_-based system. Furthermore, older emission standards are higher taxed as well as diesel cars, which motivates buyers to buy more fuel-efficient, low consumption and low emission vehicles.

**Table 10:** Performance and score of vehicle tax

Performance		Score	Refs.
*Target achievement*			
(1)	In 2020, the state income from motor vehicle tax was 9.53 Bil. € in Germany (passenger cars, LDV and HDV)		[65]
(2)	Fuel efficiency and operating cost reduction are not the primary choice objective. Therefore, the effects of CO_2_-related taxing are not as effective as for commercial vehicles. (see Fig. 3)		[4]
	The staggered tax system for passenger cars taxes high-polluting vehicles more heavily than the previous linear system Older emission standards and diesel vehicles will face higher taxes as a result of this system		[81] [81]
*Cost-efficiency*			
Not evaluated due to lack of data and studies			
*Practical feasibility*			
A reason given for levying the tax is the individual use of public infrastructure and the occupation of public space while parking			[76]
Calculation based on Euro standard, engine type (diesel, gasoline, Wankel, alternative), displacement, CO_2_ emissions from test cycles			[81]
Average vehicle age increased from 7.7 years in 2005 to 8.2 years, which induced a more extended usage of vehicles and older technology (see Additional file 1: SM 15)			[54]

### Energy tax

#### Description

Energy tax applies to all energy carriers. The annual tax revenue in Germany amounts to around 38 billion euros. In the transport sector, diesel and gasoline are the most relevant. The tax per liter is about 0.47 € for diesel and 0.67 € for gas, although the carbon- and thus the energy content of diesel is significantly higher [80]. The lower cost of diesel is because, in 2003, the EU adopted a directive to standardize oil prices. To reduce the distortion of competition between industries, a possibility of "special tax treatment" was introduced for diesel [37]. The energy tax is not further evaluated here, as it was not designed as an environmental steering instrument until 2020, which changed in 2021 with the revision of the energy tax directive [80].

### CO_2_ Tax

#### Description

The CO_2_ tax is a quantity-based levy that applies to Germany's transport and heat sectors. The tax started in 2021 with a fixed price of 25€/ton and increased gradually to 55€/ton in 2025. In 2026, the tax will be transferred to a cap-and-trade system (NEHS), such as the EU ETS. In the long term, the plan is to transfer the national systems within the EU to the EU ETS [16]. Sweden and Switzerland already implemented CO_2_ tax several years ago, but as their economies, population, GDP and land area are fully different, the results of this tax type are hard to project on Germany. In Germany, the CO_2_ tax was recently implemented during the COVID-19 pandemic. The pandemic impacted fuel and energy consumption of the (road) transport sector heavily [74]. Therefore, reliable data is not available in 2022 to evaluate the impact on consumption behavior without other influences. Subsequent studies might provide further insights on the performance of the CO_2_ tax on-road transportation in Germany.

### Low emission zones

#### Description

Low Emissions Zones (LEZ) are particularly aimed at curbing air pollution from road traffic. A LEZ is a geographical zone, usually in densely populated cities, into which only vehicles meeting a certain emission standard are allowed to enter [39]. First, it trailed in Sweden in 1996, LEZs were introduced in Germany in 2008. In 2021, 57 German cities had established LEZs [70, 78]. 

The targets of LEZs can be summarized as follows:

- Limit air pollution to
- a yearly average of 40 mg/m^3^, a daily average of 50 µg/m.^3^ for particles bigger than 10 nm (> PM10).
- a daily average may not exceed more than 35 days per calendar year.
- yearly average NO_x_ 40 µg/m^3^ [70].

#### Performance and score

Pestel et al. (2019) investigated the effect of LEZ in Germany on the nearby hospitalization for respiratory diseases related to air pollution from traffic. The authors compared the number and severity of illnesses in hospital catchment areas before and after the introduction of LEZs. The study found that:

- Hospitals with catchment areas located in an environmental zone (LEZ) diagnose significantly fewer air pollution-related diseases.
- Air quality improved considerably by reducing NO_2_ and PM10 concentrations [10].
- Improvement of public health, mainly by reducing the incidence of chronic diseases of the circulatory and respiratory systems.
- Traffic volumes and traffic-related diseases (stress, injuries) were not affected by environmental zones.
- 46 Bil. € for diseases of the circulatory system, making them the most expensive type of disease with 2.9 million cases.
- Reductions in the incidence of diseases of the circulatory system may directly reduce society's healthcare costs [58].

Results from Euro standards 1–3, whether the diesel ban including Euro 4–6 would yield any further health improvement must be researched [58]. Analysis of data from 26 monitoring stations, after correction for changes measured at background stations and traffic stations outside the environmental zones, respectively, showed a decrease in pollution of 2.1 µg/m^3^ and 2.4 µg/m^3^ for fine particulate matter (PM10) and 3.7 and 1.2 µg/m^3^ for NO_2_ as an annual average.

Margaryan et al. (2021) found that LEZ led to a 3% decline of PM10, while NO_x_ showed an insignificant reduction. The number of patients with cardiovascular disease declined by 2–3%, strongly for those aged above 65. Back-of-the-envelope cost–benefit analysis suggests health benefits of nearly 4.43 billion Euro that have come at the cost of 2. 3 billion Euro for vehicle upgrading [57]. Similar results were found in other studies [3, 39, 51].

In the following, the performance of key aspects for the selected criteria is shown in Table 11.

**Table 11:** Evaluation of low emission zones

Performance		Score	Refs.
*Target achievement*			
(1)	10–12% less particulate matter, equivalent to 20 fewer exceedance days		[70, 78]
	Two-thirds of close city measurement stations exceed NO_x_ limits		[70, 78]
	Nearby hospitalization data show a reduction of patients and respiratory diseases		[58] [57]
*Cost-efficiency*			
Cars are equipped with a sticker indicating emission class. Missing sticker or wrong sticker fine is high: 80 €			[78]
Savings in social costs are greater than upgrade costs by factor two			[3, 39, 51, 57]
*Practical feasibility*			
LEZ help to increase the share of vehicles with stricter emission standards due to access restrictions			
Costs for measurement and implementation are relatively high			[57]

### Truck road toll

#### Description

The Truck Toll is a distance-based road usage charge exclusively for heavy-duty vehicles in Germany. The toll was introduced in 2005 and represented a system change from tax-based to user-based financing of the national trunk road network. In 2020, the state income from the toll amounted to 7.4 Bil. € [8] Alternatively, the charge can be time-based (vignette), as applied in Austria.

The targets of the truck toll can be summarized as follows:

1. Shifting freight traffic to the railways (relief effects on the trunk roads, positive ecological effects, economic strengthening of the railways) [40, 69].
2. A gradual toll reduction for electric and natural gas vehicles (Federal Highway Toll Act) [8].
3. Helping to reach CO_2_ reduction targets in the transport sector [36].
4. A shift of investment costs from the state to the user (polluter-pays principle) [40].

#### Performance and score

In the following, the performance of key aspects for the selected criteria is summarized and detailly shown in Table 12.

- Set targets of the German truck toll are mostly achieved. While the total transport performance increased, the transport performance of road and rail transportation expanded as well. Road transportation increased by 28.2% and rail transport by only 17.4%, which led to a decrease in rail transport share and did not meet the goal of shifting from road to the more environmental-friendly rail (see Additional file 1: SM 14 and SM 16). Implementation of a toll supports low-carbon technologies by freeing or partly charging those technologies. In other cities (e.g., Milan), a truck toll effectively achieved a CO_2_ emissions reduction [12].
- The truck toll applies the polluter-pays principle, successfully shifted road infrastructure investment costs, and reduced state expenditure by 80%. However, the toll collection costs account for 15% of the total revenue.
- Except for the complex and cost-effective collection system implementation, the truck toll is practically feasible. Social and industrial acceptance is given due to the fact that this toll directly finances tax discounts and improved infrastructure. Furthermore, road tax reductions for low-carbon trucks help speed up transitions, reduce vehicle trips and rebound effects.

**Table 12:** Performance and score of the truck toll

Performance		Score	Refs.
*Target achievement*			
(1)	Rail share decreased from 21% (2005) to 19% (2019)		[72]
	Total transport performance increased by 17.4% from 2005 to 2019, road transportation gained 19.3%, rail gained 28,2%		[72]
(2)	The toll charge is calculated with regards to the emission standard, noise standard, gross vehicle weight of trucks—supports new technologies on the road		[8, 68]
	Zero- and low-emissions trucks receive at least a 50% discount or are free of charge to support implementation in EU countries. In Germany, these trucks are toll-free		[68]
(3)	Studies show ex-post measured CO_2_ reductions—if applied in cities—of 33% in Milan		[12]
	Existing studies illustrate that if road tolls are high, they can reduce CO_2_ emissions by 2–13% and vehicle travel by 4–22%		[12, 61]
*Cost-efficiency*			
(4)	Toll revenue: around 7.5 Bil. €		[5]
	Expenditure related to the collection of truck tolls: around 1.1 Bil. € (15% of total revenue)		[5]
	Expenditure on harmonization measures: around 537 Mil. € (incl. compensation for loss of vehicle tax revenue—7.6% of total revenue)		[5]
	Expenditure covered by truck tolls for construction, maintenance and operation of federal trunk roads: around 6.6 Bil. € (79% of road infrastructure plan)		[5]
	Road Infrastructure plan around 8.4 Bil. €		[17]
	A couple of years of operation grant an economic return on investment		[12]
	A shift of the investment costs for road infrastructure to the users		
*Practical feasibility*			
Studies show that trucks that prefer free roads pay more overall compared to toll roads. (due to congestion, traffic lights, and others.). Using tolled roads is both more environmentally friendly and cheaper			[20]
Road tax reductions for low-carbon trucks help to speed up transitions, reduce vehicle trips and rebound effects, and encourage haulers to switch to low-carbon modes of transportation			[6]
Industrial and social acceptance can be gained by tax discounts or returns as well as improvements in infrastructure and alternative mobility			[12]
Complex and cost-intensive implementation (tracker necessary for all trucks; tracking devices along the roads)			

## Final consideration

This study evaluated applied environmental policy instruments in the German road transportation sector until 2021. A wide range of instruments was applied to enhance air quality, reduce fuel consumption, and mitigate emissions. Findings demonstrate that non-market-based instruments constitute the preferred application for producers and distributors, whereas market-based instruments are chosen for consumer regulation. Furthermore, the study found:

- The effectiveness of single instruments is hard to assess and separate from other instruments, as some are aiming for the same goals
- Efficiency measures—partly induced by direct regulations—toward more efficient engines and vehicles can have a rebound effect, leading to more demand, traffic and emissions
- Measures to shift the mode of transport were not pursued consistently—the number of vehicles and transport performance steadily increased

Although governments are setting the regulations, the influence of regulators on the transportation sector is somewhat limited. A regulator cannot control the total transport development. Therefore, specific emissions can be reduced by implementing measures (e.g., fleet limits), but economic growth or side- and rebound effects might lead to higher transportation performance (e.g., more driven kilometers, traffic volume), nullifying the improvement from an emissions perspective. Other underlying reasons are changes in purchase power and processes of social change.

- Some of the criteria can only be applied to the respective instruments to a limited extent. For example, the focus on CO2 effectiveness shows that some instruments do not have a direct CO2 reduction target but have an indirect effect, such as the Euro standard.
- Furthermore, the quantitative efficiency of Euro norms and vehicle tax cannot be evaluated due to a lack of research.
- Qualitative assessment of efficiency is also difficult, as the costs and benefits are incurred in different places that are not directly offset against each other. This study prefers the macroeconomic view of costs to the microeconomic perspective.
- While microeconomic costs are relevant for companies and individuals to implement a policy, macroeconomic costs are crucial for using mechanisms and instruments. Since EPIs pursue societal goals, such as environmental protection, CO2 reduction, and air quality, the countervailing macroeconomic costs must be considered first.
- Even though the transport sector has not shown a reduction in emissions, the impact of emission-increasing developments—such as higher transport capacities, heavier vehicles and more power—has been mitigated through introduced policies.

### Non-market-based instruments

The results identify several characteristics of non-market-based instruments. Performance standards and technology mandates are popular direct regulations in the road transport sector (FQD, Euro Norm, CO_2_ fleet limit, directive on mobile air conditioning systems).

Direct regulation is difficult to implement but can take effect very quickly and is helpful for urgent problems. Thus, direct regulation has been used in the Montreal Protocol for CFC Mitigation or emissions standards (Euro Norms). However, NMBIs are very static by nature, as they are designed for specific circumstances and, therefore, do not adapt well to change (e.g., technological innovations). Moreover, they require the regulator in charge to reasonably predict technical and environmental conditions. Regulatory measures often have legislative gaps and are very complex when it comes to covering a large field. This property makes these measures—once established—resistant to innovation for alternative problem-solving.

A regulatory measure initially appears as a simple solution to a problem, but it entails a series of significant issues, revisions and adjustments. However, the effectiveness of direct interventions is undisputed. Still, economic efficiency suffers from the intervention and severely limits the ability of the regulated group to act, thus reducing the potential for alternative and innovative solutions.

The market often relies on government assistance, as seen in the transportation transition. Direct interventions—such as technology mandates—can be critical in planning government investments in necessary infrastructure. For example, the development of a charging station infrastructure, which precedes the (indirect) technology mandate of battery–electric mobility, stands in competition with developing a hydrogen refueling station network from an investment perspective. However, as governments face a limited budget and both paths call for intense investment, the regulator must decide. This decision, however, significantly limits producers’ and customers’ choices.

### Market-based instruments

Market-based instruments, especially emissions trading, represent economically more efficient approaches and do not restrict the range of solutions to the same extent. However, a fundamental problem of economic evaluation arises here:

The market and its imperfections:

As markets only cover a specific area of economic and private behavior and interactions with nature, an incomplete representation of reality arises. Thus, external effects which have not yet been internalized—i.e., not monetized and thus not appearing in the market system—do not affect the decision-making of market participants.

History has shown that many relevant external effects on the economy and environment are not internalized in market systems. For example, social costs created from respiratory diseases related to air pollution from fossil fuel burning in power plants and the mobility sector are not represented in the fuels’ cost structure. Other consequences are decreased quality of life, lower life expectancy, or loss of workforce due to induced illness by air pollution. As those effects are hard to internalize, governments decided to directly regulate air pollution (Euro standard and low emission zones). Furthermore, transport performance is not related to its eco-efficiency, which means high-emission vehicles can have the same transport performance but cause more air pollution. These costs only occur partly as higher fuel costs. The consumers’ perspective only shows higher fuel consumption and, therefore, higher transport costs, but these do not represent the actual occurring costs. As a result, many effects occur as social costs and remain public.

External effects, which affect markets, are called missing markets. Therefore, the scopes of the existing markets must be expanded to provide a sufficient representation of actual behavior and its effects (currently occurring as externalities and social costs).

An example of this is the ban on internal combustion vehicles. This mandate is an inefficient solution from an internalized market perspective as there are cheaper ways to reduce CO_2_ (low-hanging fruit principle). However, harmful emissions to health are not priced in existing cost structures yet. Therefore, the occurring costs on the health system are not represented in this price, leading to an insufficient representation. Consequences are the reduced quality of life, costs of treatments and lost work due to respiratory illnesses. Insufficient internalization of costs can be found in various energy policy examples in Germany (coal mining in the Ruhr area, nuclear power).

### How much of the present can the future take?

The economic view causes tension to arise between cost-efficiency indicators and generational fairness. There is a dilemma between static and dynamic efficiency: On the one hand, static efficiency is most relevant to align with the current economic situation and represents economic values like the return of investment or liquidity. Due to market imperfections, even static efficiency is not representing the actual costs of behaviors and might lead to a wrong path from a long-term perspective. Static efficiency does not include social costs, as those occur in the future. On the other hand, dynamic efficiency can represent these effects, but it is hard to predict, as future costs are highly uncertain. Static and dynamic efficiency can be weighed against each other using the interest rate and inflation. Thus, the return on investment of static efficiency is lower from the perspective of (future) dynamic efficiency. Furthermore, cost-efficiency indicators are different from micro and macroeconomic perspectives, leading to tension between governments and market participants.

### Internalization of climate change—CO_2_ as a determinant

Internalizing external effects is crucial for a realistic—or at least sufficient—market representation of behaviors and economic actions. Regarding the market representation of climate change effects, CO_2_ is ideally suited as an evaluation system for a spectrum of external effects. The concept of the global warming potential can convert the climate impact of different (atmospheric) gases into a single unit—CO_2eq_—which is reflected in the market via a price, thus combining these systems. However, the internalization of CO_2_ has some limitations:

- The instrument mainly focuses on emissions from fossil fuels. A complete representation of emissions will require a holistic assessment, such as the cradle-to-grave approach.
- Time preference: Time preference describes how a resource used today is valued compared to the ability to use the same resource in the future. Assuming that the CO_2_ budget is limited until 2050 or the end of the century, CO_2_ becomes a resource that can only be "used up" to a certain extent. How the budget is used up over time presents the dilemma between current and future consumption.
- The pricing scheme in emission trading systems still does not reflect emissions' time preference, as CO2 pricing in emissions trading is statically formed.
- The comparison of current and future use of resources can be represented in a CO_2_ interest rate. Thus, it can be argued that CO2 valuation shows a similar behavior with similar problems as a currency.
- Therefore, it can be argued that CO2 as the currency of our climate—when it is sufficiently developed and other externalities are internalized—could be the market-side representation of human interaction with nature.

### Outlook

As long as a market system does not reflect all—or at least the most relevant—external effects of economic trade, a purely market-oriented approach via emissions trading are neither effective nor sufficiently reflects reality.

Therefore, internalizing other relevant external effects is desirable and leads to more market-oriented approaches, openness to solutions, and a reduced necessity for direct regulations. A realistic monetary representation is complicated in some cases (quality of life, happiness, health). Whether all relevant external effects can be realistically mapped on the market side at all borders remains a technical, if not a philosophical question. In conclusion, with regard to the study results, it can be claimed that there seems to be a historical development from direct regulations to a more market-oriented search for solutions using market mechanisms, such as competition and efficiency enhancement. Integration of CO_2_ into the market system allows comparing emissions to other cost factors, with the CO_2_ price reflecting the weighting of this cost factor in the overall bill.

Even though the actual costs of today's emissions are not yet fully reflected, the market systems are improving by internalization, and it is important to encourage this development. Nevertheless, the support of the market system by regulatory measures will continue to be necessary, mainly if market actions cause external effects that are hard to monetize.

We recommend studying and assessing the transport policy after 2021, with a special focus on heavy-duty transportation, EURO 7, and alternative powertrains/fuels. Furthermore, we recommend taking life-cycle assessment into account for comparison of different powertrains and not only focusing on operative emissions.

## Supplementary Information

**Additional file 1.** European road transport policy assessment: a case study for Germany.

## Data Availability

All data generated or analyzed during this study are included in this published article (and its Additional files).

## Abbreviations

ATS: Active (Green) Technology Support
Bil.: Billion
CAC: Command-and-control
CFC: Chlorofluorocarbon
CMM: Climate monitoring mechanism
CNG: Compressed natural gas
CO_2_: Carbon-dioxide
e.g.: Exempli Gratia (for example)
EED: Energy efficiency directive
EGR: EGR exhaust gas recirculation
EPI: Environmental policy instruments
ETS: Emissions trading scheme
EU: European Union
EUETS: European Union’s Emissions Trading Scheme
EV: Electric vehicle
FQD: Fuel quality directive
GHG: Greenhouse gases
ICCT: International Council of Clean Transportation
IPCC: Intergovernmental Panel on Climate Change
LED: Light-emitting diode
LEED: Leadership in Energy and Environmental Design
LEZ: Low-emission zone
MBI: Market-based instruments
Mil.: Million
NEDC: New European drive cycle
NMBI: Non-market-based instruments
NOAEL: No observed adverse effect level
NOx: Nitrogen-oxide
OBD: On board diagnostics
PM: Particle matter
RD&D: Research, development and deployment
RDE: Real driving emissions
RED: Renewable energy directive
ROI: Return of investment
SCR: Selective catalytic reduction
SM: Supplementary material
SUV: Sport utility vehicle
TWh: Terawatt hours
UNFCCC: United Nations
UNFCCC: United Nations framework convention on climate change
VECTO: Vehicle energy consumption calculation tool
WLTP: Worldwide harmonized light vehicles test procedure
