# Supplementary material for: European road transport policy assessment: a case study for Germany
Source: Environ Sci Eur. 2022 Sep 15;34(1):92. doi: 10.1186/s12302-022-00663-7 (PMC9476439; doi:10.1186/s12302-022-00663-7)
Supplement: Supplementary file 1 — Additional file 1. European road transport policy assessment: a case study for Germany. [file 12302_2022_663_MOESM1_ESM.docx]

**IUE**

**Hamburg University of Technology**

**Institute of Environmental Technology and Energy Economics**

**Supplementary Material**

**Policies in Germanys Road Transportation –**

**Evaluation of Mechanisms and Effects**

Hamburg, May 2022

# Table of Content

[Table of Content 1](#_Toc103269528)

[Figures and Tables 2](#_Toc103269529)

[1. General Principles 2](#_Toc103269530)

[1.1. Sustainability Principle 3](#_Toc103269531)

[1.2. Precautionary Principle 3](#_Toc103269532)

[1.3. Prevention Principle 4](#_Toc103269533)

[1.4. Polluter Pays Principle 4](#_Toc103269534)

[1.5. Burden Sharing Principle 5](#_Toc103269535)

[1.6. Low-Hanging Fruit 6](#_Toc103269536)

[1.7. Eco-efficiency principle 6](#_Toc103269537)

[1.8. Extended Producer Responsibility 6](#_Toc103269538)

[1.9. Interactions of Environment, Markets, Regulations and Environmental Policy Instruments 6](#_Toc103269539)

[2. Environmental Policy Instruments 9](#_Toc103269540)

[2.1. Market-Based Instruments 9](#_Toc103269541)

[2.1.1. Taxes 9](#_Toc103269542)

[2.1.2. Emissions Trading Schemes 10](#_Toc103269543)

[2.1.3. Removal of Perverse Incentives 12](#_Toc103269544)

[2.1.4. Liability Instruments 12](#_Toc103269545)

[2.1.5. Deposit-Refund Systems 13](#_Toc103269546)

[2.2. Non-Market-Based Instruments 13](#_Toc103269547)

[2.2.1. Command-and-Control 13](#_Toc103269548)

[2.2.2. Reporting Requirements 13](#_Toc103269549)

[2.2.3. Active Green Technology Support 14](#_Toc103269550)

[2.2.4. Removal of Green Tech Financial Barriers 14](#_Toc103269551)

[2.2.5. Information and Voluntary Approaches 14](#_Toc103269552)

[2.3. Road Transport Assessment Statistics 16](#_Toc103269553)

[2.3.1. Specific emissions from passenger cars 16](#_Toc103269554)

[2.3.2. Detailed Evaluation of passenger car taxation in Germany 16](#_Toc103269555)

[2.3.3. HDV Figures: 18](#_Toc103269556)

[2.4. Road Transport Assessment Statistics 21](#_Toc103269557)

[2.4.1. Greenhouse Gas Emissions 21](#_Toc103269558)

[2.4.2. Final Energy Demand 22](#_Toc103269559)

[2.4.3. Transport Performance 22](#_Toc103269560)

[2.4.4. Vehicle Fleet 23](#_Toc103269561)

[3. References 25](#_Toc103269562)

# Figures and Tables

[SM 1: Interactions of Environment, Markets, Regulator and Environmental Policy Instruments 37](#_Toc95734030)

[SM 2: Specific emissions from passenger cars over time [115] 38](#_Toc95734031)

[SM 3: Car taxation system in Germany in 2021 based on [130] 38](#_Toc95734032)

[SM 4: Car vehicle tax in Germany in 2021 for cars registered after 2021. Cost of displacement by vehicle type (left) and CO2 -Emissions for 2020 and 2021 (reformation), own graphic based on [130] 39](#_Toc95734033)

[SM 5: Comparison of the car fleet in EU28 and Germany in 2019 by fuel type [1] 40](#_Toc95734034)

[SM 6: HDV tax system in Germany in 2020. (S2, S1: Emission standards, G1: Noise standard), own graphic based on [130] 41](#_Toc95734035)

[SM 7: Development of Euro standards in HDV fleet in Germany from 2010 to 2021, data from [76] 41](#_Toc95734036)

[SM 8: Commercial vehicle fleet Germany 2020: All fuel types vehicle weight (left); alternative fuels in commercial vehicle fleet by vehicle weight (right), data from [30] 42](#_Toc95734037)

[SM 9: Baseline and accelerated policy timeline for light-duty vehicles [20] 42](#_Toc95734038)

[SM 10: Timeline of greenhouse gases from sectors in Germany and share of transport sector [118] 43](#_Toc95734039)

[SM 11: Timeline of greenhouse gases from the transport sector in Germany and transport emissions target until 2030 [118] 43](#_Toc95734040)

[SM 12: Timeline of final energy of sectors in Germany [55] 44](#_Toc95734041)

[SM 13: Timeline of final energy demand in transport sector [55] 44](#_Toc95734042)

[SM 14: Transport Performance and mode share in Germany between 2013 and 2019 (own figure) [99, 103] 45](#_Toc95734043)

[SM 15: Fleet age of passenger cars, light-duty and medium- and heavy-duty vehicles in 2019 [1] 46](#_Toc95734044)

[SM 16: Transport performance of goods transportation modes in Bil. ton-kilometers [116] 46](#_Toc95734045)

# General Principles

Environmental policy is driven by several underlying background principles, which are implemented in Germany and other nations' environmental laws:

## Sustainability Principle

The concept of sustainability was developed in the 17^th^ century by Hans Carl von Carlowitz based on the idea of sustainable forestry. Following the principle, the amount of cut-down trees must equal the amount of natural regeneration of trees. The famous Brundtland report from 1987 by the United Nations contains the currently accepted definition of sustainability: [34]

"Humanity has the ability to make development sustainable – to ensure that it meets the needs of the present without compromising the ability of future generations to meet their own needs."

Nowadays, sustainability is the guiding concept on which 17 sustainable development goals are created. Economic perspective categories the sustainability principle in two major types, strong and weak sustainability. Constanza and Daly (1992) created two rules for decision-making with imperfect knowledge to maintain "natural capital":

1. Renewable source consumption is limited to sustainable yield levels.
2. Revenue of non-renewable resource exploitation is invested into renewable natural capital.

Based on this perspective, renewable natural capital and human-made capital are complements rather than substitutes. For example, a refinery is useless without oil. This category is called strong sustainability. Weak sustainability, on the other hand, defined by the Hartwick rule, claims that the total amount of natural and human-made capital is constant over time. The weak concept defines the two types of capital as substitutes.

Furthermore, the idea of critical natural capital describes sustainability as close to zero and a harmful irreversible imbalance between natural and human-made capital. So far, there is no answer on the exchangeability of human-made and natural capital. [11] This idea leads to another question: How far are the negative effects of exploiting natural capital monetizable in human-made capital? This question has a significant impact on environmental policy choice and, in particular, on market-based instruments.

## Precautionary Principle

The precautionary principle is a common principle of health and environmental policy, enacted to address irreversibility and imperfect knowledge of negative effects of present behavior. [2] Therefore, the prevention of environmental damage has a higher priority than removing it. It is derived from the sustainability principle to prevent irreversible damage to the environment and keep options open for future generations. Especially when environmental costs of economic behavior are uncertain, and we are seriously concerned about future generations, precaution is the only "sensible strategy." [9] The world charter in 1982 defined it as "where potential adverse effects are not fully understood, the activities should not proceed." The most significant problem of enforcing this principle is various interpretations of itself. From an economic point of view, the definition of the principle is vague, and interpretation is left to current regulators in charge, which might be used as a blurred form of trade protectionism. Furthermore, the principle can hinder innovations. [11] Environmental policy instruments contained the precautionary principle from an economically asymmetric manner, as undershooting the emission mitigation target is not allowed, whereas overshooting is appreciated. [9]

Therefore, the Rio declaration stated in 1992 that the absence of total scientific certainty should not hinder the deployment of cost-effective measures to prevent environmental degradation. [11] However, applying the principle is not easy when potential damage occurs in the future, mainly when the cost for protection is high and immediate. Prevention of potential threats necessitates action before scientific evidence of consequential damage. [9]

## Prevention Principle

The Prevention Principle is similar to the precautionary principle but differs in terms of knowledge about the potential environmental consequences and impacts of economic behavior. [11] The Prevention principle can be applied if negative effects and consequences of economic activity are partly known and understood. [2] As detailed knowledge about possible consequences is hard to achieve, the precautionary principle is more often applied. Therefore, the precautionary principle is more extensive than the prevention principle.

## Polluter Pays Principle

The polluter pays principle focuses on making consumers and producers responsible and is aware of their behavior by pricing - environmentally harming - externalities. The key is internalizing negative external effects by embedding the cost of negative impacts throughout pricing the consumption. [42] The principle is based on the idea that the pollution itself and the consequential environmental damage is unavoidable. The principle that should be embedded in environmental law is described in the Kyoto Protocol under principle 16. [64]

Therefore, market-oriented instruments are introduced to avoid environmental pollution and promote resource efficiency. Policy-makers design mechanisms – based on the polluter principle ­­– to ensure that polluters take responsibility for the entire damage costs of their activities through liability provision, regulations, taxes, and others [27, 42]. Those mechanisms try to bring the private and social costs of economic behavior closer together while maintaining the freedom to market participants to find economically efficient alternatives (see technological openness/bias). The challenge for regulators is to find commensurate prices Risk and impact assessment are available measures to determine external effects and create fair prices. [27] Pricing schemes are potential market distortions, which can create unintentional effects like carbon leakage. [64]

Regulators can influence economic behaviors by internalizing external effects in the market system. Then, the resource use will be dependent on the pricing scheme and the price elasticity of producers and consumers (i.e., willingness-to-pay). [42]

A necessity for enacting polluter pays is that the identifiable otherwise, the social costs are borne by the burden-sharing through society. It has become an important background principle for many industrialized countries in environmental policy. The main driver for this development was adopting this principle by the Organization for Economic Cooperation (OECD) in 1972, followed by the European Union in 1985. Nevertheless, the polluter pays principle induced many issues, which are summarized by Boorsma et al.: [7]

- "What really is an externality, and how should we define the costs associated with it?
- For precisely which control measures should the polluter be accountable?
- Which norms should guide us here? On what basis should payments, if any, be determined and implemented?
- And, continuing, should the specification of the polluter pays principle include, besides the costs of (preventive) measures, damage costs too?
- And should polluters under such an "extended" polluter pays principle (OECD, 1994) also pay for pollution below the levels that are at present thought to be compatible with an acceptable state of the environment (because the environmental capacity to absorb wastes is limited)?
- Furthermore, it was soon realized that imputing full social costs for various reasons often may neither be socially nor politically acceptable. What, for example, if the implementation of the principle would substantially increase unemployment in less-developed regions?
- What if a polluting factory or industry is deemed important for strategic purposes?"

The principle is applied in the Piguo Tax, where Pigou declares that neglecting social cost leads to significant market distortion. In summary, there are two options for using the polluter pays principle: First, by implementing direct or non-market-based regulations based on standards or permits. Second, the introduction of market-based instruments like charges, penalties, or compensation duties in proportion to the emissions. In practice, the polluter pays principle is extremely hard to implement. [7]

As regulators seek to avoid environmental damage and incentive resource conservation based on the prevention and precautionary principle, it was advocated to take the user into account. Other types of polluter-pays principles were derived: The **polluter and user-pays principle**. [7]

Further, the implications of the welfare of countries join this discussion, in which the 'rich countries pay for the actions of 'poor' ones. This principle is called the **victim pays principle**, which may be engaging in terms of the European Union, where central Europe might pay for the actions of eastern European countries. For example, the European Union financially supports Poland to replace its coal-based power capacity with renewable energies. Another example is a wastewater treatment plant, which is subsidies by the government. [67]

## Burden Sharing Principle

Burden sharing is a fairness principle – like polluter pays – which includes egalitarian aspects which focus on the ability to pay for achieving goals. It does not impose equal sharing of resources but includes cost-sharing aspects, which are crucial for political acceptance within a society, but especially on a global scale. [8]

## Low-Hanging Fruit

The low-hanging fruit principle describes a cooperate strategy, which refers to the easiest, most obvious, or most cost-effective investments available to achieve a certain goal. On the one hand, following the strategy can lead to meeting goals rapidly, but on the other hand, it can lead to the accumulation of more difficult problems. [28]

## Eco-efficiency principle

Eco-efficiency concepts contrast the destruction of ecological values with the creation of economic value. It enables to measure products and processes from an ecologic and economic standpoint and the comparison with competitors and markets. [24] Therefore, it monitors the ecological effects at every stage of production to optimize with the least possible use of resources while keeping the same or higher outcome. [65] Improvements – like technical innovations – can be measured with the eco-efficiency and compared to alternatives. Examples of application are the World Business Council for Sustainable Development and BASF's eco-efficiency concept [24, 29]

## Extended Producer Responsibility

The OECD describes the extended producer responsibility as a principle in which the responsibility for a product life-cycle is enlarged to the post-consumer stage. Therefore, the physical and/or financial responsibility is shifted upstream toward the producer to incentive environmental-friendly production processes. [64]

## Interactions of Environment, Markets, Regulations and Environmental Policy Instruments

The producing market requires inputs in the form of resources to create value. Within the market boundaries, these resources are used in value chains and, in simplified terms, leave the market as outputs. The effects resulting from the use of these inputs within an overall value chain till they are output can be divided into internal and external effects. Figure 3 shows the interactions of the market with the environment and a regulator using environmental policy instruments (EPI).

- Internal effects represent the impact of value creation and trade within the market. They are in interaction with the market participant and form – depending upon formation – an equilibrium. The internal effect of the market participant is thus presented to him directly and is incurred as a cost.


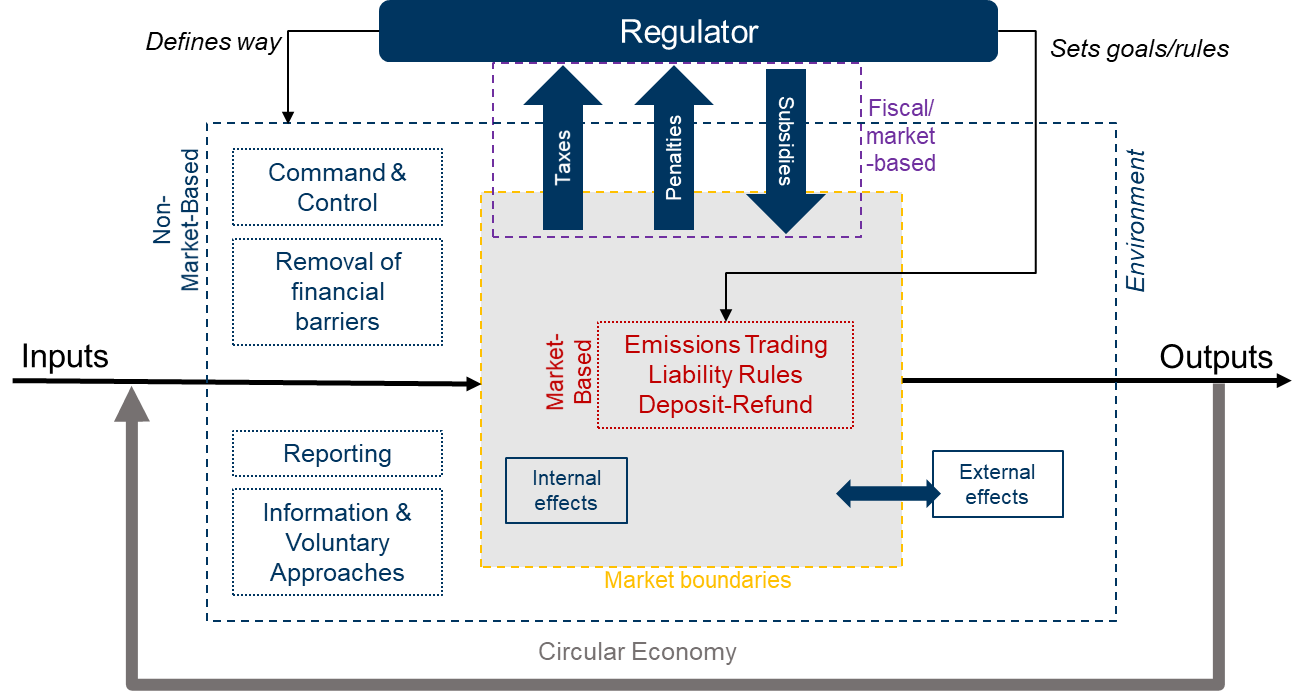


SM 1: Interactions of Environment, Markets, Regulator and Environmental Policy Instruments

- External effects are also generated by trading within the market but are not reflected in the market and thus do not lead to a balance between market-side trading and externalities. Therefore, the market can continue to trade undisturbed without perceiving any effects in the context of its metrics in order to contain trading. External effects, however, interact with the market and usually show up in another area of the market, accrue as social costs of current or future generations. These effects usually remain hidden from the tempter.

To mitigate the effects of externalities, a regulator can use environmental policy instruments. These EPIs are applied at different points in the market and to the market participants. According to the subdivision into market-based and non-market-based EPIs, a regulator can exert control on trading.

- First, the regulator can apply market-based fiscal instruments, such as taxes, penalties and subsidies (bonus-malus). This approach follows the polluter-pays principle. This involves a two-way exchange between the regulator and the market participants.
- Second, a regulator can install market-based instruments within the market and define the necessary rules for market participants to use the instruments. Once the rules are set, market participants can continue to act under these new conditions. A new balance is establishing itself in the market, which should cause fewer external effects. This approach also follows the polluter-pays principle.
- Third, a regulator can define the framework, rules and goals for using non-market-based instruments. These influences from the outside on the market try to reduce the external effects by restricting the market. This approach follows the precautionary or preventive principle. From the market's point of view, this results in market distortion.

All environmental policy instruments must be carefully designed to adequately reflect the externalities arising from the actions of market participants.

# Environmental Policy Instruments

Environmental policy instruments (EPI) are categorized into market- and non-marked-based instruments. Figure 1 shows the main instruments for climate policy which are described in more detail in the following sections. The different instruments are not always clearly distinguishable from each other by definition. The designed mechanisms often appear as gradations and combinations of the various instrument types presented here. Underlying principles of EPIs are described in supplementary material 6.2. The interaction of environment, markets, regulations and environmental policy instruments is further described in supplementary material 6.3.


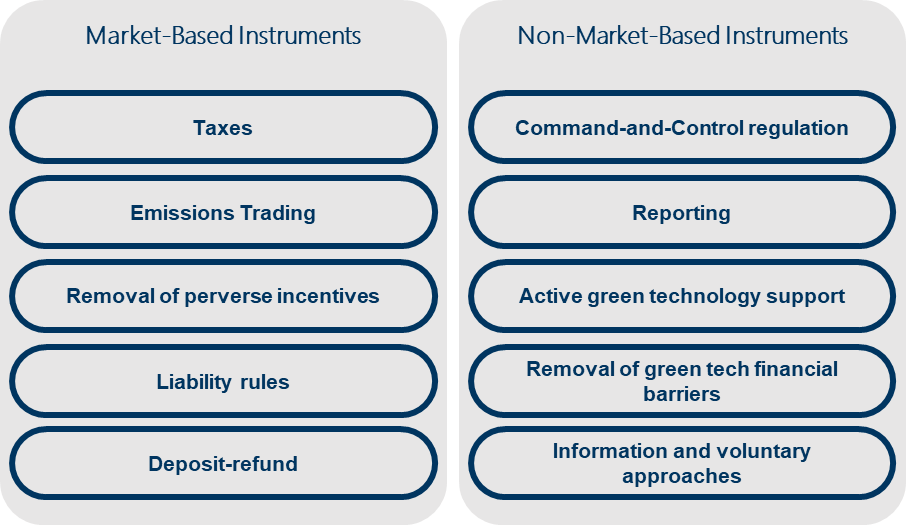


Figure 1: Overview of environmental policy instruments, based on [26, 53]

## Market-Based Instruments

Market-based instruments utilize the “polluter pays principle” by confronting polluters with the costs of their pollution (Supplementary Material 6.2). The respective information (here: costs caused by the pollution) is needed [64]. But often, the real cost for the consequences of a particular behavior is not represented within the market (i.e., market imperfections, missing markets) because these costs usually accrue as external effects to economies and/or can hardly be quantified in a sound and transparent way (e.g., costs of climate change). So far, these costs burden the public even though individuals are not responsible for these emissions. By internalizing the costs of such external effects, the polluter pays for its behavior. In terms of CO_2_ emissions, specific CO_2_ taxes and CO_2_ trading systems have been developed as essential instruments of such market-based measures to imply explicit internalization. Possible penalties of direct regulations achieve implicit internalization due to non-compliance [64].

### Taxes

In an environmental manner, a tax and charges system should be imposed on the manufacturer and the user, representing the external costs of their actions, following the price control mechanism [40]. The most common greenhouse gas (GHG) tax is a carbon tax or a tax for CO_2_-equivalents (i.e., based on the global warming potential of GHG) [26].

- A carbon tax should encourage the integration of low-carbon options (i.e., energy carrier and mobility technologies), making those more competitive within the respective markets, where carbon-intensive possibilities are still profitable without intervention [54].
- By taxing pollution, the legal authorities influence the markets by increasing prices. The emission quantities still released into the atmosphere are expected to depend on the price change due to these taxes and the respective price elasticity [42]. Such taxes are often put on production processes' inputs and outputs. A tax on fossil fuels, for example, does not even require active monitoring of the respective CO_2_ emissions because the legislator must not link this tax to a particular reduction goal (even though, in most cases, they do). Other cases are the imposition of taxes on product specifications (e.g., fuel efficiency) or activities (e.g., climate tax on airline tickets). Other forms are financial support mechanisms like a differential tax rate or direct support for, e.g., “green” energy carriers (direct payments, subsidized loans) [26].

High taxes might restrict private decision-making and negatively affect savings and investment, thus influencing economic growth [26]. In this case, the carbon price is specified and determined by the regulation. The consequence is that a certain tax's absolute CO2 emission reductions are unknown beforehand [7, 26].

The Border Adjustment Tax is a special type of tax meant to be applied at the border of an economic area to protect its economy from – mostly cheaper – imports and prevent the leakage of domestic industries due to lower-cost production environments. Carbon leakage describes the outflow of industries and production capacities to foreign countries without or less carbon pricing. In terms of CO_2_, carbon-border adjustment is a mechanism to respond to carbon leakage and create fair conditions for – carbon price strained – industries. Similar systems are already used to adjust different value-added tax systems of countries. [53] In terms of the Green Deal of the EU, the Carbon Border Adjustment Mechanism (CBAM) will be implemented to address carbon leakage. [18]

### Emissions Trading Schemes

Emissions trading schemes or systems use market mechanisms to reduce emissions instead of distorting the market via taxes or financial benefits. Two different systems are applied (Figure 2). Those systems require reporting about actual emissions (see chapter 2.2.2).


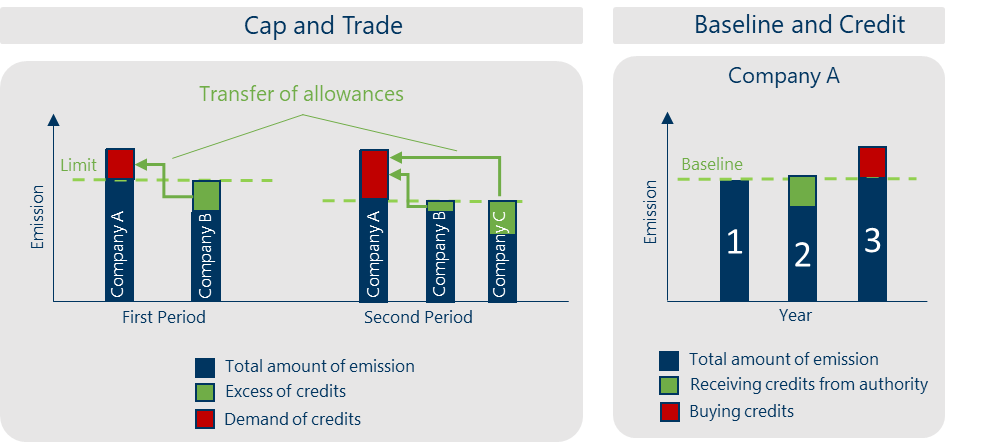


Figure 2: Types of emissions trading schemes, based on [35]**.** Left: Cap and Trade. Right: Baseline and credit.

**Cap-and-trade** systems limit the overall amount of a given pollutant, following a quantity control mechanism [45]. The regulator imposes a defined market limit in a defined trading period, often characterized by a decreasing slope for the periods to come. Afterward, the regulator allocates (free) pollution permits to the market participants for a specific price or free of charge, following a pre-defined mechanism (e.g., defining an emissions cap for a particular technology). Market participants can now trade these pollution allowances, which might make CO_2_ emission savings profitable for companies [26]. According to Figure 2 (left), company A exceeds the limit for its company in the first period, and it has to buy certificates from another company. Meanwhile, company B´s and C´s emissions are below their limit. Therefore, those companies B and C can sell their allowances to company A.

By the end of each trading period, each company needs to have sufficient allowances for their emissions released into the atmosphere during the respective trading period. In the second period, the limit is lowered by the regulator. Should a company not have reduced its emissions, it must buy even more allowances from other companies. The price of these allowances is formed similar to a stock exchange by supply and demand.

Market stability reserves (MSR) are an additional tool for regulators to withdraw or add allowances to stabilize prices. For example, if there is a vast amount of excess allowances in the market, the price is stably low and creates no incentive to transform towards a system with lower GHG emissions for a long time. Under these circumstances, the regulator takes allowances out of the market with the possibility of putting them back into the market if necessary (e.g., if the prices are rising too fast) [22]. Such regulation can include the entire market, specific sectors or particular industries (the European Emissions Trading Scheme (EUETS), for example, includes the energy and industrial sector [5]).

**Baseline-and-credit** systems require a minimum performance obligation to a predefined baseline emissions profile. The regulator sets the limit for every participant. These emission reductions create tradeable allowances to be sold to other market participants exceeding their baseline. Such systems do not set upper limits. Therefore, overall emission reduction is not inevitably given [26]. Figure 2 (right) shows a constant baseline for a company for three years. In the first year, the company exactly meets the limit. In the second year, the company falls below its limit and receives credits from the regulator, which the company can sell to other companies exceeding their limits. In the third year, the company exceeds its limit and must buy allowances from other companies. If the trading period is set for several years, a company can balance its allowances within those years. Such systems are usually used for individual sectors, while cap systems are more likely to be applied cross-sectorally.

**Smart or flex cap systems** determine price and quantity along a particular supply curve for allowances. This so-called "FlexCap adjustment function" determines the number of allowances auctioned depending on the price. If the price is high, more allowances are auctioned, and the cap increases. If the price is low, fewer allowances are auctioned, and the cap decreases. Both adjustments stabilize the price compared to an emissions trading system with a fixed cap but do not keep the price constant like a tax system. Economic literature largely agrees that uncertainty about marginal (abatement) costs leads to exceptionally high costs when fully passed on to consumers and firms or low prices without any impact. [52] Therefore, smart or flex cap systems are hybrid quantity and price control systems.

| 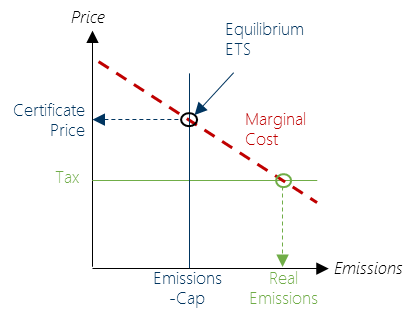 | 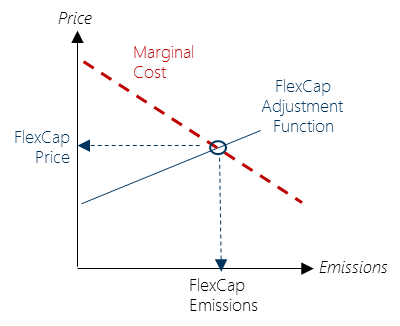 |
| --- | --- |

Figure 3: Relations of marginal cost, emissions and price for tax and Emission Trading Schemes (ETS) – cap and trade – (left) and FlexCap (right).

Figure 3 shows the interactions of marginal cost, emissions and price for tax, Emissions Trading Schemes (ETS) and FlexCap. The red dotted line represents the marginal (abatement) costs (e.g., CO_2_ abatement costs). The difference between quantity control and price control is shown in the left figure. In the case of quantity control, the budget of a resource is fixed, and the price is set by the marginal (abatement) cost. In the case of price control, a fixed tax determines the real emissions according to the marginal (abatement) cost. The right figure shows the flex cap approach. A regulator sets a flex cap adjustment function, which creates a relation between price and emissions.

### Removal of Perverse Incentives

Some subsidies might be hazardous to the environment since they encourage pollution or the use of energy-intensive products or operations [20]. For example, many developing and emerging countries subsidize fossil fuel consumption by keeping the market price artificially low [26]. Thus, several OECD countries want to decrease subsidies and protections favoring specific sectors or industries, especially those that negatively affect the environment, while simultaneously imposing costs in the form of taxes or allowances [38]. Examples of liability framework corrections include exclusions from responsibility or incomplete liability regulations, creating an implicit subsidy and distorting competition [26].

### Liability Instruments

Liability rules push companies to consider the environmentally harmful effects of their commercial activities [26, 51]. Since no technologies or practices are defined a priori by such instruments, a liability rule can theoretically be a cost-effective policy instrument. Imposing financial responsibility on hazardous products and their disposal, for instance, encourages companies to minimize their use of those materials. In addition, the proof is very administratively intensive. However, it does not provide overall incentives for companies to decrease societal dangers associated with those materials [43]; an example is the liability of nuclear power plants [26].

By adapting the existing liability framework to new technologies, clear liability rules implemented by the regulator should give manufacturers, service providers and operators the confidence to assess their liability and obtain appropriate insurance. Without modified liability rules, injured parties would have to pay for the cost of the damage [21].

### Deposit-Refund Systems

A deposit refund system combines an excise tax with a refund when the product or packaging is returned for recycling or proper disposal [59]. Such a deposit-refund strategy effectively addresses pollution by imposing an upfront tax on production or consumption and using the fee revenue to reimburse environmentally friendly inputs and remedies [6].

Many deposit refund programs are implemented within the waste management industry. The most innovative ones realize an "upstream" approach. They do not offer refunds to consumers who return products to recycling collection sites but subsidize collectors who deliver materials to re-processors or the re-processors themselves if they convert the collected material into usable secondary materials [60].

## Non-Market-Based Instruments

### Command-and-Control

Command-and-Control (CAC), also 'direct or implicit regulation,' is a direct regulatory intervention. Command-and-Control (CAC) is often used for pollution control or managing common property resources (e.g., overfishing). Instead of a complex monetary valuation of environmental effects, physical units measure the benefits [11, 64]. This tool is used to set limits and standards in terms of climate policy, usually for CO_2_ emissions or energy efficiency. It is implemented and enforced in regulations, often combined with different penalties for non-compliance [26, 41]. Command-and-Control (CAC) policies address two major types: technology mandates and performance standards [9].

**The regulator sets technology mandates or technology standards** to phase out or ban specific production processes and equipment from utilization that harms the environment. One example of enforcement is the EU *EcoDesign Directive* providing rules for environmental performance, particularly products' energy efficiency. This directive regulates the standby and off-mode of devices and – for example – enforces the replacement of traditional light bulbs with LED [26, 41]. Command-and-Control (CAC) technology mandates were also used within the Montreal Protocol prohibiting the production of chlorofluorocarbons (CFCs) [3, 32, 37].

**Performance standards** (minimum energy performance standards or benchmarks) provide more flexibility than technology mandates, aiming at specific environmental goals instead of banning or dictating technologies [9]. Typical examples are fleet average CO_2_ vehicle efficiencies; nitrogen oxides (NO_x_) or particulate matter (PM) emissions are regulated with this instrument. For example, a specific CO_2_ emissions limit of 95 g_CO2_/km in 2020 has been defined for new-registered passenger cars within the EU and further decreased until 2030 [4, 9, 14, 26]. Another example is building codes and standards-setting standards for construction and energy use [26].

### Reporting Requirements

Reporting requirements (also stand-alone reporting) help track economic and environmental behavior and create a base for future regulation, legislation, or other (non-market- or market-based) instruments. It is used as a first step to assess the status quo, collect data, increase the level of information, and/or as a basis for emissions trading systems [26]. Additionally, reporting requirements help to enhance awareness of pollutants. Collecting data creates a consciousness of “what goes through the chimney” [41]. In the EU, regulation requirements are implemented in the Climate Monitoring Mechanism. This instrument accompanies the European Energy Efficiency Directive (EED) as well as the Renewable Energy Directive (RED) [17]; furthermore, it helps to keep track of member states' emission targets [26].

### Active Green Technology Support

Active green technology support (ATS) promotes green technologies' development, adoption, and deployment[41, 45] by improving their availability and artificially making them more cost-effective for a market. ATS's emphasis directly impacts shifting supply to greener technologies instead of relying on environmentally friendly demand [45].

Active technology support as an instrument has typical market elements involved but is usually strongly regulated and connected to specific technologies or behaviors. Therefore, it is categorized as a non-market instrument [41]. Such policies include public and private RD&D funding, public procurement, green certificates, renewable portfolio standards, feed-in tariffs, and public investment in infrastructure [45]. An example is a financially supported development and the following cost reduction for solar PV. Quota obligations are also part of this instrument, like bioethanol's utilization and commitment to fuels (e.g., E5 and E10).

Further, feed-in tariffs for private solar PV deployed ATS in Germany (see EEG [55]). A feed-in tariff is a guaranteed price for selling a good for a specific timeframe to provide investment security and a predictable investment return. Public infrastructure investments can help lower the financial barriers that households and industries face when acquiring green technologies [26].

### Removal of Green Tech Financial Barriers

Mechanisms to remove financial barriers for green tech help to mobilize private capital for investment in exclusively environmental-friendly technologies [13, 26]. This instrument refers to the demand-side to support the deployment of products and processes. It is realized via subsidized loans, capital allowance, tax breaks and reduction, as well as direct payments [41]. Financial barriers for green tech are, e.g., uncertainties and higher risks, lack of information, hidden costs, limited access to capital, split incentives and bounded rationality [33]. Even though these instruments adopt economic incentives, they focus on incentivizing specific technologies, making them tightly regulated and not open to market flexibility [41]. One example of removing financial barriers is the 'Ecobonus' for purchasing electric vehicles for customers.

### Information and Voluntary Approaches

Information and voluntary approaches influence the behavior of societal actors by addressing their moral sense and changing a cultural environment [41]. Private or industrial voluntary approaches can be caused by shifting public opinions about products and behaviors. Such information might increase customers' awareness about the environmental impact of products and provide information on less harmful alternatives [97]. The information framework provided by this instrument does not directly regulate products, processes, and behaviors. Instead, this instrument addresses not only a level of logic at the receiver but also a level of ethics, morals, or emotions.

Types of information and voluntary approaches are product certification and labeling, voluntary agreements, unilateral commitments, award schemes, public information campaigns, as well as public voluntary schemes [41].

- *Product certification and labeling* is an instrument that simplifies information transfer to customers of products and indicates adherence to specific standards. In addition to voluntary labels, there may also be labels prescribed by the legislator. Independent third parties often create these labels but can also be related to industrial organizations following a particular marketing goal. Typical examples are energy efficiency labels (mandatory in the EU), LEED certification (voluntary) for buildings, or the Blue Angel for environmentally friendly products in Germany [26].
- *Voluntary agreements* between governments and industrial companies or entire sectors aim to lower harm to the environment. Voluntary agreements allow industry partners to negotiate conditions and keep flexibility [97] while simultaneously reducing bureaucracy for the government in creating and enforcing laws. Monitoring, strictness, and sanctions are negotiated and differ among these agreements [97].
- *Unilateral commitments* are instruments for companies and industry groups to commit to specific environmental goals voluntarily (like carbon neutrality) [26]. Typical goals are self-imposed emission reductions to claim carbon neutrality within a definite timeframe. Voluntary agreements and unilateral commitments can prevent the industry from direct regulation instruments.
- *Award schemes* draw public attention to environmental accomplishments by a public board. Under a set of criteria, performance is assessed and awarded on a national and international level (e.g., "European Business Awards for the Environment" [16]).
- *Public information campaigns* are organized by public actors or groups (e.g., Greenpeace) to increase awareness and pressure governments and industry groups regarding environmental topics and concerns [26]. These campaigns can lead to public movements and demonstrations.
- *Public voluntary schemes* refer to the voluntary adoption and deployment of standards, targets, and reporting for companies created by public authorities or industry groups. Information is open to the public and provides a high degree of transparency [41]. For example, the eco-management and audit scheme provides reporting templates about the company's environmental activities [19].

# Road Transport Assessment Statistics

## Specific emissions from passenger cars

SM 2: Specific emissions from passenger cars over time [56]

## Detailed Evaluation of passenger car taxation in Germany

- Tax system until 2009 based on engine displacement, engine type, and Euro standard - the poorer the exhaust quality, the higher the tax per 100 cubic centimeters or part thereof of engine capacity
- Reformation of the tax system in 2009 – integration of CO_2_-emissions from test-cycle as cost-driver
- According to the 2020 report "CO_2_ -Based Motor Car Taxes in the European Union," 24 EU nations levy vehicle taxes that are partially or entirely based on CO_2_ emissions and fuel usage. [15]
- In France, Germany, and the UK, the tax gap between CO_2_ and high CO_2_ vehicles is relatively low between 2014 and 2018, whereas the Netherlands and Norway offer better four-year tax breaks. [62]
- The VAT rate was reduced from 19 percent to 16 percent in the second half of 2020, which lowers the acquisition costs for low-emission vehicles in general. A one-off premium for electric vehicles, on the other hand, drastically reduces acquisition costs. [25]
- There has been no significant improvement in diesel emissions from newly manufactured vehicles. This is because actual carbon emissions from new diesel cars have fallen by only 8% since 2000, and actual carbon emissions from new light commercial vehicles have fallen by only 2%.[63]

SM 3: Car taxation system in Germany in 2021 based on [66]

| Registration | Until 2009 | 2009 - 2021 | from 2021 |
| --- | --- | --- | --- |
| Euro Standard | Euro 0-3 | not included | not included |
| Engine Type | Gasoline,  Diesel | Gasoline,  Diesel | Gasoline,  Diesel |
| Engine Displacement  [per 100cm^3] | G: 6.75€ - 25.36€ D: 15.44€ - 37.58€  (determined by Euro standard) | G: 2€ D: 9.5€ | G: 2€ D: 9.5€ |
| CO_2_ - Emissions  (NEDC/WLTP) | not included | 2 €/gCO_2_ above limit | 2 - 4 €/gCO_2_ above limit |
| CO_2_ - tax exemption limit [gCO_2_/km] (registration date) | not included | till 2012: <120g/km till 2014: <110 g/km from 2014: <95 g/km | 2021: <95 g/km |

(G: Gasoline; D: Diesel)

SM 3: Car taxation system in Germany in 2021 based on [130] summarizes the car taxation system in Germany in 2021. The taxation of cars was reformed several times, which led to a graduation of the taxation after the first registration. In 2021, cars registered before 2009 will be assessed according to the Euro standard, fuel and engine capacity. Registrations between 2009 and 2020 will also be assessed according to specific CO_2_ emissions, increasing linearly.

Cars registered from 2021 onwards will be assessed according to a staggered, slightly exponentially increasing cost curve for CO_2_. This reform leads to relatively higher taxation of cars with higher CO_2_ emissions. (see SM 4)

Furthermore, there is a CO_2_ exemption limit for cars below which no taxes have to be paid. This mechanism is intended to support market entry for low- or zero-emission vehicles and make them more economically attractive. SM 4 illustrates the car vehicle taxation in Germany in 2021. Diesel vehicles are taxed more heavily than gasoline vehicles based on engine displacement since diesel vehicles are responsible for a large share of inner-city emissions. 65% of NO2 emissions in urban areas come from diesel cars, 12% from truck traffic (mostly diesel), and 16% from other commercial vehicles. Only 3% of NO_x_ emissions are from vehicles using other fuels. [63]

|  |  |
| --- | --- |
| SM 4: Car vehicle tax in Germany in 2021 for cars registered after 2021. Cost of displacement by vehicle type (left) and CO_2_ -Emissions for 2020 and 2021 (reformation), own graphic based on [66] | |

In a study conducted in France, Germany and Switzerland, the effects of vehicle taxes on CO_2_ emissions were examined. It shows that although the effect is higher in France, where CO_2_-based purchase taxes and subsidies have been introduced, the taxes have a significant negative impact on new vehicle registrations in the short term in all countries. The study used annual vehicle taxes that increased in proportion to carbon dioxide emissions. However, they could not detect a clear indication of manufacturers' long-term commitment to low-emission cars. [36]

The impact of the new vehicle tax on vehicle choice has not yet been determined. The introduction of a penalty system for high-polluting vehicles, similar to the promotion of low-emission vehicles, could also help to co-finance higher purchase incentives for electric vehicles [61].

SM 5: Comparison of the car fleet in EU28 and Germany in 2019 by fuel type [1]

SM 5 demonstrates the EU28 and German car fleet in 2019 by fuel type. The share of diesel cars in Germany is lower than in the EU28 by 10.6%. However, diesel cars are still prevalent in Germany because of several incentives: company cars, lower fuel prices due to lower energy taxes and lower fuel consumption because of higher efficiency and higher energy content of diesel. However, Germany's number of diesel cars rose by 25.2% between 2011 and 2021 due to the (indirect) incentives. [47]

Different levels of vehicle taxation can strongly influence buyers' decisions. If taxation is adequately designed, the conversion of vehicle fleets to low-emission vehicles could accelerate. [62] Member states may use tax incentives under certain conditions (e.g., if the former incentive expires when new limits take effect) to accelerate the introduction of instruments that meet the new standards before the statutory deadline. [12] Many European countries have directed their incentives to buy zero or low emission vehicles, yet they have implemented the vehicle taxation scheme that supports high emission vehicles. [62] There are conflicts between the tax system and policy. The reasons for these discrepancies are insufficient purchasing information between expensive technology and low fuel costs, friction in the used car markets, inappropriate incentives in the markets for company cars, and manufacturers' uncertainty about how car buyers will react to more efficient but expensive vehicles. [39] The diesel scandal has created enormous momentum and changed public perception. However, it is more advantageous in many European countries to own and use a diesel vehicle than other fuel types or even other transportation modes. [44]

## Heavy-Duty Vehicle Figures:

HDV: Operational costs are the main cost driver in the total cost of ownership. As commercial vehicle fleet owners (e.g., haulers) tend to minimize costs (rational decision-maker), market participants look for potential cost savings in operation costs. Therefore, fuel savings – hence CO_2_ savings – are the main optimization objective. [31]

SM 6: HDV tax system in Germany in 2020. (S2, S1: Emission standards, G1: Noise standard), own graphic based on [66]

SM 7: Development of Euro standards in HDV fleet in Germany from 2010 to 2021, data from [30]

|  |  |
| --- | --- |
| SM 8: Commercial vehicle fleet Germany 2020: All fuel types vehicle weight (left); alternative fuels in commercial vehicle fleet by vehicle weight (right), data from [30] | |


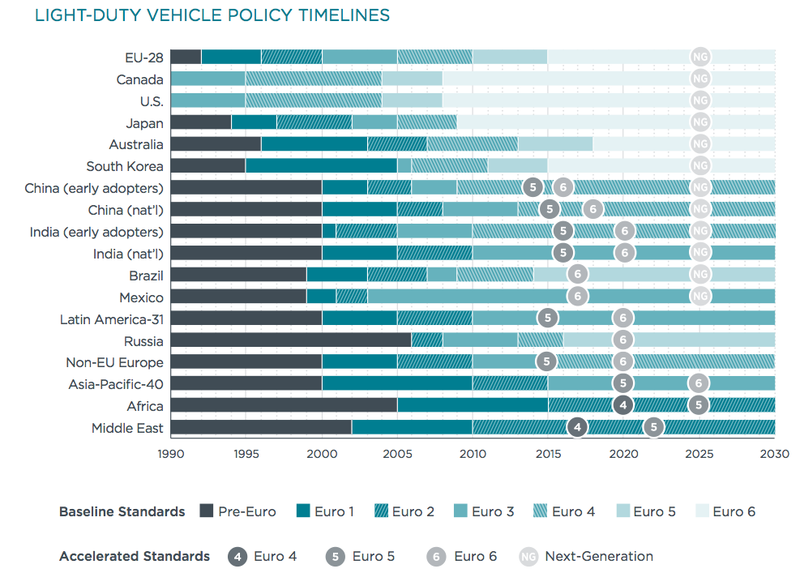


SM 9: Baseline and accelerated policy timeline for light-duty vehicles [10]

## Greenhouse Gas Emissions

SM 10: Timeline of greenhouse gases from sectors in Germany and share of transport sector [58]

SM 11: Timeline of greenhouse gases from the transport sector in Germany and transport emissions target until 2030 [58]

## Final Energy Demand

SM 12: Timeline of final energy of sectors in Germany [23]

SM 13: Timeline of final energy demand in transport sector [23]

## Transport Performance

Heavy-duty road transportation is the dominant freight transport mode in Germany. The share rose from 70.4% in 2013 to 72.5% in 2020. In the same year, the freight transport performance of rail was 18%, inland shipping 6.9%, and pipeline 2.5%. All these transport modes – except for road transport – are predicted to decrease until 2024 because the German government continues to rely on road transport, judging by the planned investments in transport infrastructure. In 2021, the freight performance share of road transport slightly decreased – due to the COVID-19 – and is predicted to increase to 72.7% by 2024. [50]

SM 14 illustrates the transport performance and mode share of the road, rail, inland shipping and pipeline transport in Germany between 2013 and 2019. While the total transport performance increased, the share of transport remained nearly constant, around 72%.

SM 14: Transport Performance and mode share in Germany between 2013 and 2019 (own figure) [46, 50]

## Vehicle Fleet

Europe 2019:

- 97.8% Diesel Trucks
- 42.3% Diesel passenger cars, 42.3% Petrol; 2.7% LPG, 0,4% Bev & PHEV
- 569 cars per thousand habitants

Germany 2019:

- 97.9% Diesel Trucks
- 31.7% Diesel passenger cars, 66% Petrol; 0.8% LPG, 0,5% Bev & PHEV
- 569 cars per thousand habitants

Diesel is fueling the German heavy-duty fleet. In 2021 about 93.4% were diesel trucks, 4.4% petrol, and 2.2% were other fuel types. In particular, medium and heavy-duty trucks are 99.5% diesel (2019) in Germany [1, 49]. Further, the average age of duty vehicles in Germany is 8.2 years in 2021 (2019: 7.7 years) [48]. Especially, the average age of heavy-duty vehicles is 9.5 years in Germany, in Europe 13 years [1]. It is notable that – due to lower vehicles taxes – heavy-duty vehicles are mostly registered in other European countries like Poland or Italy. As the emission standards data of HDV is hardly available in these countries, this study focuses on the impacts of EPI on the German fleet.

SM 15: Fleet age of passenger cars, light-duty and medium- and heavy-duty vehicles in 2019 [1]

SM 16: Transport performance of goods transportation modes in Bil. ton-kilometers [57]

References

[1] ACEA. 2021. *Vehicles in Use*. *Report* 23. European Automobile Manufacturers Association.

[2] Balzer, C. 2017. *Vorlesung Umweltbewertung*. *Umweltrecht*.

[3] Baumann, S., Elsner, C., Graaf, D. de, Hoffmann, G., and Martens, K. *1987 - 2017: 30 Jahre Montrealer Protokoll*. *Vom Ausstieg aus den FCKW Vom Ausstieg aus den FCKW zum Ausstieg aus teilfluorierten zum Ausstieg aus teilfluorierten Kohlenwasserstoffen Kohlenwasserstoffen*.

[4] BMU. 2019. *Die EU-Verordnung zur Verminderung der CO2 - Emissionen von Personenkraftwagen*.

[5] BMWi. 2020. *Wie funktioniert eigentlich der Europäische Emissionshandel?* https://​www.bmwi-energiewende.de​/​EWD/​Redaktion/​Newsletter/​2020/​08/​Meldung/​direkt-erklaert.html. Accessed 3 August 2021.

[6] Bohm, P. 1983. Deposit-Refund Systems: Theory and Applications to Environmental Conservation and Consumer Policy. *The Scandinavian Journal of Economics* 85, 3.

[7] Boorsma, P. B., Aarts, K., and Steenge, A. E. 1997. *Public Priority Setting: Rules and Costs*. Springer Netherlands, Dordrecht.

[8] Bretschger, L. 2013. Climate policy and equity principles: fair burden sharing in a dynamic world. *Environment and Development Economics* 18, 5, 517–536.

[9] Buchholz, W. and Rübbelke, D. 2019. *Foundations of Environmental Economics*. Springer International Publishing, Cham.

[10] Chambliss, S., Miller, J., Facanha, Cristiano, Minjares, Ray, and Blumberg, K. 2013. *The impact of stringent fuel and vehicles standards on premature mortality an emissions*. *ICCT report finds global implementation of advanced emissions and fuel-quality regs could cut early deaths from vehicle emissions by 75% in 2030*. https://​www.greencarcongress.com​/​2013/​11/​20131106-icct.html.

[11] Common, M. S. and Stagl, S. 2005. *Ecological economics*. *An introduction*. Cambridge University Press, Cambridge, UK, New York.

[12] DieselNet. 2021. *EU: Heavy-Duty Truck and Bus Engines*. *Regulatory Framework*. https://​dieselnet.com​/​standards/​eu/​hd.php.

[13] EPA. 2008. *Removing Market Barriers to Green Development*. *Principles and action projects to promote widespread adoption of green development practices*.

[14] Europäische Union. 2019. *VERORDNUNG (EU) 2019/ 631 DES EUROPÄISCHEN PARLAMENTS UND DES RATES - vom 17. April 2019 - zur Festsetzung von CO2-Emissionsnormen für neue Personenkraftwagen und für neue leichte Nutzfahrzeuge und zur Aufhebung der Verordnungen (EG) Nr. 443/ 2009 und (EU) Nr. 510/ 2011*.

[15] European Automoble Manufacturers' Assocaton. *Overview – CO2-based motor vehicle taxes in the European Union – ACEA – European Automobile Manufacturers' Association*.

[16] European Commission. 2020. *About the European Business Awards for the Environment (EBAE)*. https://​ec.europa.eu​/​environment/​awards/​about_ebae.html. Accessed 14 October 2020.

[17] European Commission. 2020. *Emissions monitoring & reporting*. https://​ec.europa.eu​/​clima/​policies/​strategies/​progress/​monitoring_en. Accessed 13 October 2020.

[18] European Commission. 2020. *EU Green Deal (carbon border adjustment mechanism)*. https://​ec.europa.eu​/​info/​law/​better-regulation/​have-your-say/​initiatives/​12228-EU-Green-Deal-carbon-border-adjustment-mechanism-_en.

[19] European Commission. 2020. *What is EMAS?* *The EU Eco-Management and Audit Scheme (EMAS)*. https://​ec.europa.eu​/​environment/​emas/​index_en.htm. Accessed 14 October 2020.

[20] European Commission. 2021. *Environmental Economics*. https://​ec.europa.eu​/​environment/​enveco/​mbi.htm.

[21] European Commission. 2021. *Inception Impact Assessment*. *Adapting liability rules to the digital age and circular economy*.

[22] European Commission. 2021. *Market Stability Reserve*. https://​ec.europa.eu​/​clima/​policies/​ets/​reform_en. Accessed 28 September 2021.

[23] Eurostat. 2021. *Energy Balance 2021*. *Germany*. https://​ec.europa.eu​/​eurostat/​de/​web/​energy/​data/​energy-balances. Accessed 4 August 2021.

[24] Gabler Wirtschaftlexikon. 2021. *Ökoeffizienz*. *Definition: Was ist "Ökoeffizienz"?* https://​wirtschaftslexikon.gabler.de​/​definition/​oekoeffizienz-52330. Accessed 21 July 2021.

[25] 2020. *Germany’s vehicle tax system: Small steps towards future-proof incentives for low-emission vehicles*.

[26] Görlach, B. 2013. *What constitutes an optimal climate policy mix?* *Defining the concept of optimality, including political and legal framework conditions*. Ecologic Institute, Berlin.

[27] Harremoës, P. 2001. *Late lessons from early warnings*. *The precautionary principle, 1896-2000*. Environmental issue report no. 22. European Environment Agency, Copenhagen Denmark.

[28] Hayes, A. 2021. *Low-Hanging Fruit*. https://​www.investopedia.com​/​terms/​l/​low-hanging-fruit.asp. Accessed 21 July 2021.

[29] Kaltschmitt, M. and Schebek, L. 2015. *Umweltbewertung für Ingenieure*. *Methoden und verfahren / Martin Kaltschmitt, Liselotte Schebek, herausgeber*. Springer Vieweg, Berlin Germany.

[30] KBA. 2021. Fahrzeugzulassungen (FZ). Bestand an Kraftfahrzeugen nach Umwelt-Merkmalen. 2010 - 2021.

[31] Ketterer, J., Wackerbauer, and Johann. 2009. *Die Kraftfahrzeugsteuer als Instrument der Klimaschutzpolitik* 4. Leibniz-Institut für Wirtschaftsforschung an der Universität München e. V.

[32] Knauer, R. *25 Jahre FCKW-Verbot*. *Es bleibt ein Loch ohne Boden*. https://​www.spektrum.de​/​news/​25-jahre-fckw-verbot-wie-steht-es-um-das-ozonloch/​1352353.

[33] Knobloch, F. and Mercure, J.-F. 2016. The behavioural aspect of green technology investments: A general positive model in the context of heterogeneous agents. *Environmental Innovation and Societal Transitions* 21, 39–55.

[34] Lexikon der Nachhaltigkeit. 2015. *Nachhaltigkeit Definition*. https://​www.nachhaltigkeit.info​/​artikel/​definitionen_1382.htm. Accessed 27 April 2021.

[35] Miyata, Y., Shibusawa, H., and Fukuda, T. 2015. *Environmental and Economic Impact of Carbon Credit in Makassar City in Indonesia*, 55th Congress of the European Regional Science Association: "World Renaissance: Changing roles for people and places".

[36] Nadirov, O., Vychytilová, J., and Dehning, B. 2020. Carbon Taxes and the Composition of New Passenger Car Sales in Europe. *Energies* 13, 18, 4631.

[37] Naumann, M. 2019. *FCKW: Das hat das Verbot dieser Treibhausgase bewirkt*. https://​utopia.de​/​ratgeber/​fckw-das-hat-das-verbot-dieser-treibhausgase-bewirkt/​.

[38] OECD / OCDE. 2005. *Environmentally Harmful Subsidies*. *Challenges for Reform*.

[39] OECD / OCDE. 2008. *The Cost and Effectiveness of Policies to reduce Vehicle Emissions*. *Summary and Conclusions*.

[40] Pigou, A. C. 2002. *The economics of welfare*. Classics in economic series. Transaction Pub, New Brunswick NJ.

[41] Prahl, A. and Hofmann, E. 2017. *Non-Market-Based Climate Policy Instruments*. https://​climatepolicyinfohub.eu​/​non-market-based-climate-policy-instruments.

[42] Rademaekers, K. and van der Laan, J. 2011. *The role of market-based instruments in achieving a resource efficient economy*.

[43] Revesz, R. L. 2006. *Foundations of environmental law and policy*. Foundations of Law Series. LexisNexis, New Providence NJ.

[44] Runkel, M. and Mahler, A. 2018. *A comparison of CO2 -based car taxation in EU-28, Norway and Switzerland*. *Fair & Low Carbon Vehicle Taxation in Europe*.

[45] Serres, A. de, Murtin, F., and Nicoletti, G. 2010. *A Framework for Assessing Green Growth Policies. DOI=*10.1787/5kmfj2xvcmkf-en.

[46] Statista. 2020. *Transportleistung je Verkehrsträger in Deutschland von 2010 bis 2019*. *(in Milliarden Tonnenkilometer)*. https://​de.statista.com​/​statistik/​daten/​studie/​75784/​umfrage/​gueterverkehr-in-deutschland-nach-verkehrszweigen/​. Accessed 20 July 2021.

[47] Statista. 2021. *Anzahl der Personenkraftwagen mit Dieselmotor in Deutschland von 2011 bis 2021*. https://​de.statista.com​/​statistik/​daten/​studie/​251779/​umfrage/​bestand-von-pkws-mit-diesel-motor-in-deutschland/​.

[48] Statista. 2021. *Durchschnittliches Alter der Lastkraftwagen in Deutschland von 2011 bis 2021*. https://​de.statista.com​/​statistik/​daten/​studie/​154528/​umfrage/​durchschnittliches-alter-der-lkw-in-deutschland/​. Accessed 20 July 2021.

[49] Statista. 2021. *Durchschnittliches Alter der Lastkraftwagen in Deutschland von 2011 bis 2021*. https://​de.statista.com​/​statistik/​daten/​studie/​154528/​umfrage/​durchschnittliches-alter-der-lkw-in-deutschland/​.

[50] Statista. 2021. *Modal-Split im deutschen Güterverkehr im Zeitraum von 2013 bis 2024 nach Landverkehrsträgern*. *(Anteil an der Transportleistung)*. https://​de.statista.com​/​statistik/​daten/​studie/​12149/​umfrage/​gueteraufkommen-nach-verkehrstraegern-in-deutschland/​.

[51] Stavins, R. N. 2003. Experience with Market-Based Environmental Policy Instruments. In *Environmental Degradation and Institutional Responses*, J. Vincent and Mäler Karl-Göran, Eds. Handbook of Environmental Economics. Elsevier, 355–435. DOI=10.1016/S1574-0099(03)01014-3.

[52] Traeger, C., Perino, Grischa, Pittel, Karen, and Requate, T. 2019. *Das Flexcap – eine innovative CO2-Bepreisung für Deutschland*.

[53] Tsafos, N. 2020. *How Can Europe Get Carbon Border Adjustment Right?* https://​www.csis.org​/​analysis/​how-can-europe-get-carbon-border-adjustment-right.

[54] Tsafos, N. 2020. *In Defense of the Energiewende*.

[55] Umweltbundesamt. 2019. *Erneuerbare-Energien-Gesetz*. https://​www.umweltbundesamt.de​/​themen/​klima-energie/​erneuerbare-energien/​erneuerbare-energien-gesetz.

[56] Umweltbundesamt. 2021. *Emissionen des Verkehrs*. https://​www.umweltbundesamt.de​/​daten/​verkehr/​emissionen-des-verkehrs. Accessed 20 September 2021.

[57] Umweltbundesamt. 2021. *Fahrleistungen, Verkehrsleistungen und „Modal Split“*. https://​www.umweltbundesamt.de​/​daten/​verkehr/​fahrleistungen-verkehrsaufwand-modal-split.

[58] Umweltbundesamt. 2021. *Previous year's estimate of German greenhouse gas emissions for 2020*. *GHG Dataset*, Dessau.

[59] Walls, M. 2016. *Deposit-Refund Systems in Practice and Theory*. Resources of the Future. DOI=10.5194/gmd-2016-10-SC1.

[60] Walls, M. and Palmer, K. 2001. Upstream Pollution, Downstream Waste Disposal, and the Design of Comprehensive Environmental Policies. *Journal of Environmental Economics and Management* 41, 1, 94–108.

[61] Wappelhorst, S. 2020. *Germany’s vehicle tax system: Small steps towards future-proof incentives for low-emission vehicles*. https://​theicct.org​/​blog/​staff/​germany-vehicle-tax-system-sept2020.

[62] Wappelhorst, S., Mock, P., and Yang, Z. 2018. *Using vehicle taxation policy to lower transport emissions: An overview for passenger cars in Europe*. *An Overview for passenger cars in Europe*.

[63] Wehrmann, B. 2019. *Germany's environment agency says emissions of new diesel cars still too high*.

[64] Wiesmeth, H. 2012. *Environmental Economics*. Springer Berlin Heidelberg, Berlin, Heidelberg.

[65] Wuppertaler Institut. 2001. *Mehr durch immer weniger - das Prinzip Öko-Effizienz hat die Wirtschaft erreicht*. *Das Wuppertal Institut veranstaltet am 15. und 16. Mai 2001 die Konferenz auf der ENVITEC*. https://​wupperinst.org​/​a/​wi/​a/​s/​ad/​211. Accessed 21 July 2021.

[66] Zoll. 2021. *Steuerhöhe*. https://​www.zoll.de​/​DE/​Unternehmen/​Kraftfahrzeugsteuer/​Steuerhoehe/​steuerhoehe_node.html;jsessionid=AD0BC26D74B8CA1AA72A5B84CC4138EC.internet402. Accessed 17 August 2021.

[67] Zylicz, T. 2015. *The Economics of International Environmental Cooperation*. Polish Studies in Economics, Frankfurt am Main, Berlin, Bern, Bruxelles, New York, Oxford, Wien.
